# A highly selective C–H bond fluorination unlocks conformational reporting in a complex natural product derivative†

**DOI:** 10.1039/d5sc01857a

**Published:** 2025-03-26

**Authors:** Jonah Ruskin, Roxanne Dekeyser, Nathaniel Garrison, Phoebe Williams, Maya Kramer-Johansen, Ananya Majumdar, Travis Dudding, Adam Huczyński, Thomas Lectka

**Affiliations:** a Department of Chemistry, Johns Hopkins University 3400 North Charles St. Baltimore MD 21218 USA lectka@jhu.edu; b Department of Chemistry, Brock University St. Catharines Ontario L2S3A1 Canada; c Department of Medical Chemistry, Faculty of Chemistry, Adam Mickiewicz University Poznań Poland

## Abstract

The site-selective C–H bond fluorination of complex natural products is one of the more sought-after transformations in organic and medicinal chemistry. In many radical-based fluorinations, however, a tempest of poor regio- and stereoselectivity, multiple additions of fluorine, and difficult separations of products conspire to make selective monofluorination appear out of reach. In our fluorination of the antibiotic ionophore salinomycin and its simple derivatives, however, a chain of discoveries, including an unanticipated skeletal rearrangement, provided us a tortuous but unique path to a very selective result, unlocking low-noise conformational reporting by ^19^F NMR in a widely studied medicinal scaffold.

## Abstract 2

In our fluorination of the antibiotic salinomycin and its simple derivatives, a chain of discoveries provided us a unique path to a selective result, unlocking low-noise conformational reporting by ^19^F NMR in a widely studied medicinal scaffold.

## Graphical abstract

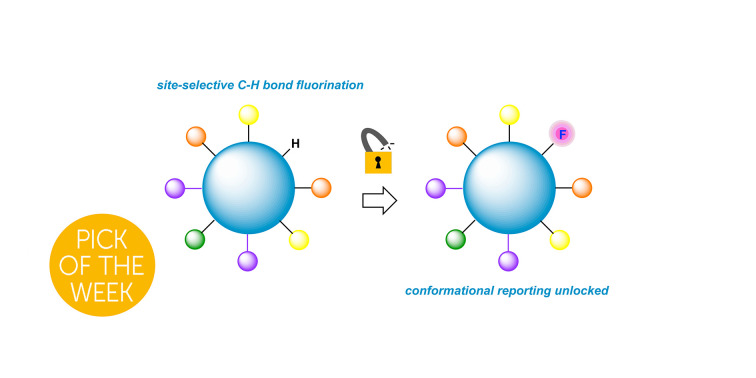

## Introduction

**Fig. 1:**
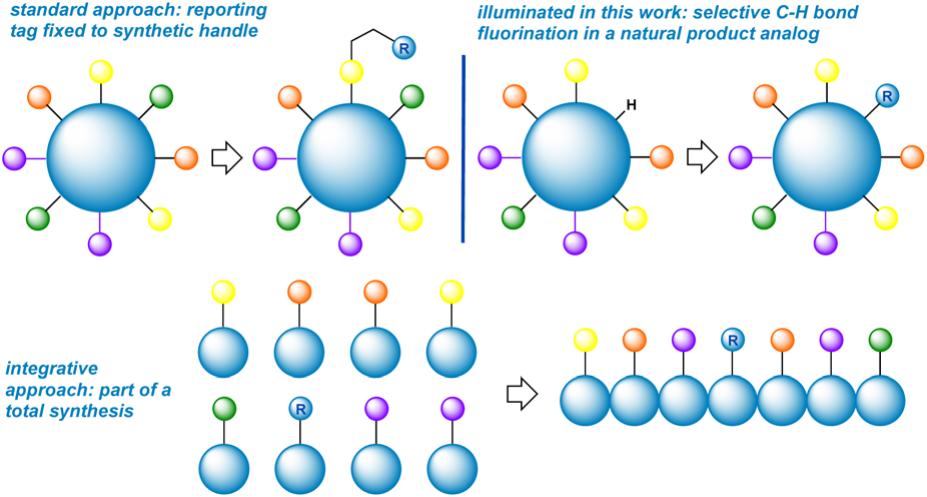
Three complementary approaches to conformational reporting.

The incorporation of fluorine “tags” into proteins and DNA has provided an elegant means for studying the conformational dynamics of large biomolecules.^1^ For comparatively small complex natural products and their analogues, however, what options remain when addition of a conformationally floppy and spatially removed peripheral tag (reporter) cannot distinguish conformers (Fig. 1)^2^ One excellent approach is to integrate the reporter as part of a fragment in a total synthesis.^3^ A virtually unexplored approach would be direct fluorination of a skeletal backbone through C–H bond activation. This would offer a creative solution by placing the reporting fluorine nucleus in close proximity to nearby substituents (Fig. 1). The installation of the least sterically demanding group (fluorine) apart from a hydrogen atom is also an attraction.^4^

**Fig. 2:**
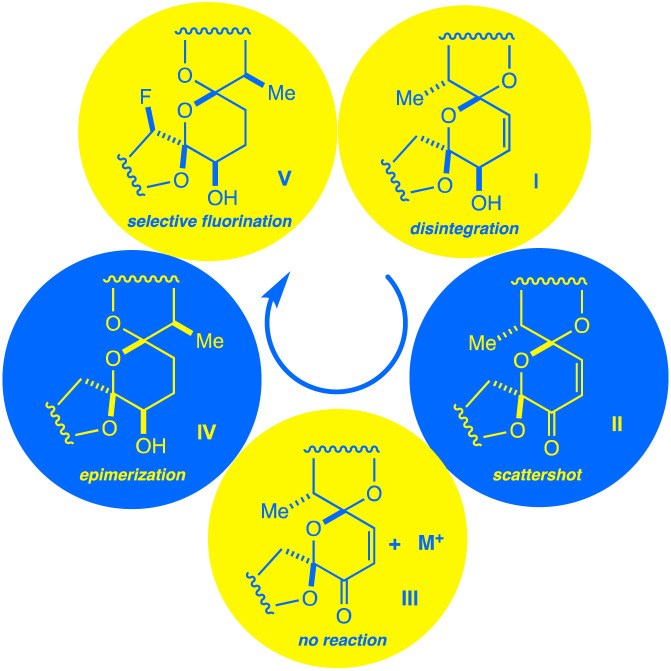
A chain of discoveries and setbacks (*I*–*V*) leads to the isolation of a monofluorinated salinomycin derivative.

Even so, the difficulty of executing a highly selective C–H bond fluorination accounts for the fact that the concept remains fairly unexplored.^5^ In our group's pursuit of natural product fluorination, however, an excellent selectivity test presented itself in the form of the complex antibiotic ionophore salinomycin (SAL, 1). Herein, we disclose a chain of discoveries leading to the highly selective fluorination of this widely studied and biologically active complex natural product scaffold (Fig. 2). While engineering a selective transformation was our primary goal, we were gratified to discover a fluorine probe that demonstrates remarkable sensitivity to chemical environment and enables facile, low-noise conformational monitoring by ^19^F NMR. In turn, we believe that site-selective C–H bond activation represents a potential third, complementary approach to conformational reporting.

Over the previous decade, synthetic and biochemical interest in SAL and its derivatives has steadily intensified.^6^ A growing collection of over 100 semisynthetic SAL derivatives has emerged as the molecule's backbone is repeatedly shown to inhibit both therapy-resistant cancer cells and cancer stem cells through metal-binding mechanisms.^7^ Importantly, the role of conformational dynamism in the SAL backbone has been a focal point in wide-ranging studies on the molecule's in-solution behavior,^8^ metal-binding ability,^9^ passage through membranes,^10^ and biological activity.^11^

In our review of the literature, however, we find that no attempts have been made to functionalize the SAL core directly through C–H activation, let alone fluorination.^7^ In fact, all fluorine-containing SAL derivatives have been synthesized by appending the SAL backbone with prefluorinated fragments.^12^ Shi and coworkers, for example, previously outfitted SAL with a fluorine tag using standard click chemistry to provide sensitivity to ^19^F MRI techniques.^2^ However, it is noted that their derivative is not found to be conformationally probative following chelation to a variety of metal ions.

At first glance, the feasibility of a site-selective C–H modification of 1 looks poor. SAL is a densely functionalized substrate featuring three ethereal rings woven into a unique bis-spiroketal ring system. To complicate matters further, this tricyclic bis-spiroketal is bracketed by additional ethereal rings as well as a straight chain region possessing free rotation.^13^ Even so, our prior experience led us to believe that selective monofluorination was worth pursuing. On the one hand, we have extensively researched the ability of directing groups on rigid substrates to activate select sites to fluorination.^14^ On the other, we have shown that the conformational changes and deactivating effects induced by chelation to a metal ion can be exploited to fluorinate a dynamic substrate with high selectivity.^15^ With both rigid and freely rotating structural features, we suspected that SAL derivatives may be compatible with our fluorination methods. We reasoned that, between our prior experience, the cache of known semisynthetic SAL modifications, the established ability of SAL to bind cations,^16^ and existing crystal structure data,^17^ we had sufficient knowledge and variables within our control to execute a fluorination of the SAL backbone.

Underpinning these efforts, Selectfluor remained our fluorinating reagent of choice. Whereas other attempts at selectively modifying the SAL backbone have involved bioengineering designer enzymes,^18^ we felt it was important to rely on commonplace and easily accessible reagents. Mechanistically, Selectfluor radical dication (SRD) is generated under previously established photochemical conditions^19^ and subsequently undergoes electrophilic hydrogen atom transfer (HAT) with the SAL substrate. Selectfluor intercepts the radical intermediate that results, fluorinating the substrate and propagating a chain reaction.

## Results and discussion

**Fig. 3:**
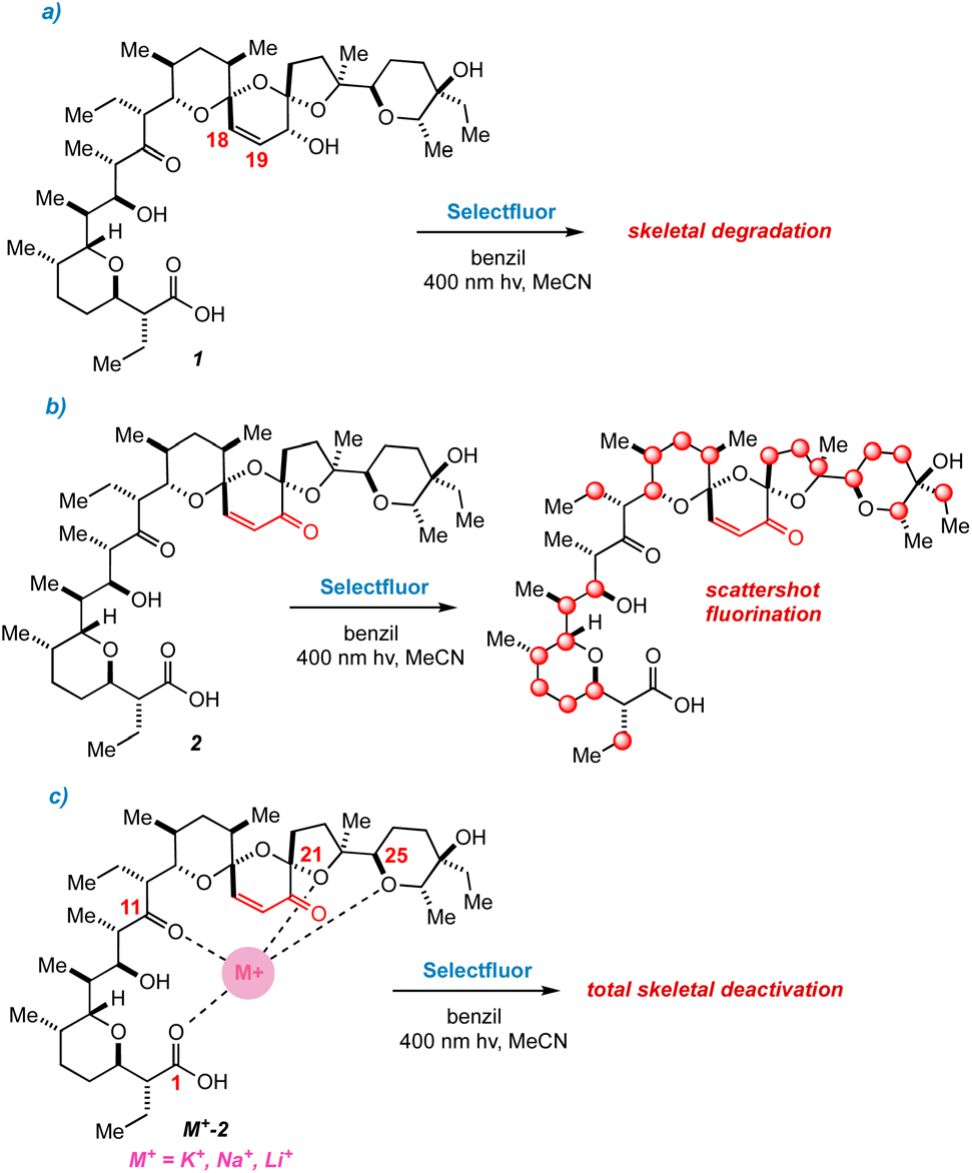
(a) Decomposition of 1 under photochemical conditions. (b) Scattershot fluorination observed for 2. (c) Metal complexes of 2 are deactivated to fluorination.

Our first major setback arrived early in the project. By simply mixing Selectfluor and 1 in acetonitrile, we found that putative single electron transfer (SET) occurs at the C_18_=C_19_ bond, thereby engendering concomitant unspooling of 1's skeleton. This phenomenon results in a messy array of aromatic degradation products that also pertained under photochemical conditions (Fig. 3a). The ability of alkenes to undergo SET with Selectfluor has featured in our previous work,^20^ so the intolerance of 1 to our method did not surprise us.

The next approach sought to solve two problems at once – namely to deactivate the C_18_=C_19_ bond and to provide a directing group to an adjacent ring.^14*c*^ Fearful that generating open-shell intermediates on the fragile C ring of SAL would result in degradation, converting 1 to the SAL enone 2 seemed to be a reasonable next step.^12*a*^ We quickly learned that this idea was also seriously misguided. Subjecting 2 to radical fluorinating conditions resulted in a multitude of products by ^19^F NMR (Fig. 3b). None of these products was created in appreciable yield for isolation, although we did note that the backbone of 2 remains stable to our reaction conditions.

In recent work, we overcame the problem of indiscriminate fluorination by deactivating large molecular fragments through metal ion chelation.^15^ Conveniently, SAL has an affinity for potassium, sodium, and lithium cations, coordinating metals mainly through O_1_, O_11_, O_21,_ and O_25_.^8,17^ Hopeful that monovalent cations would limit the available reactive sites in 2, we repeated our fluorinations in the presence of each of these metals. Suddenly we faced an entirely new issue; whereas apo-2 fluorinates with promiscuity, metal-bound 2 does not fluorinate at all, and only starting material is recovered (Fig. 3c). By deactivating the bis-spiroketal region of SAL with an enone, and chelating the remaining portion of the backbone, HAT is too disfavored to sustain a chain reaction.

**Fig. 4:**
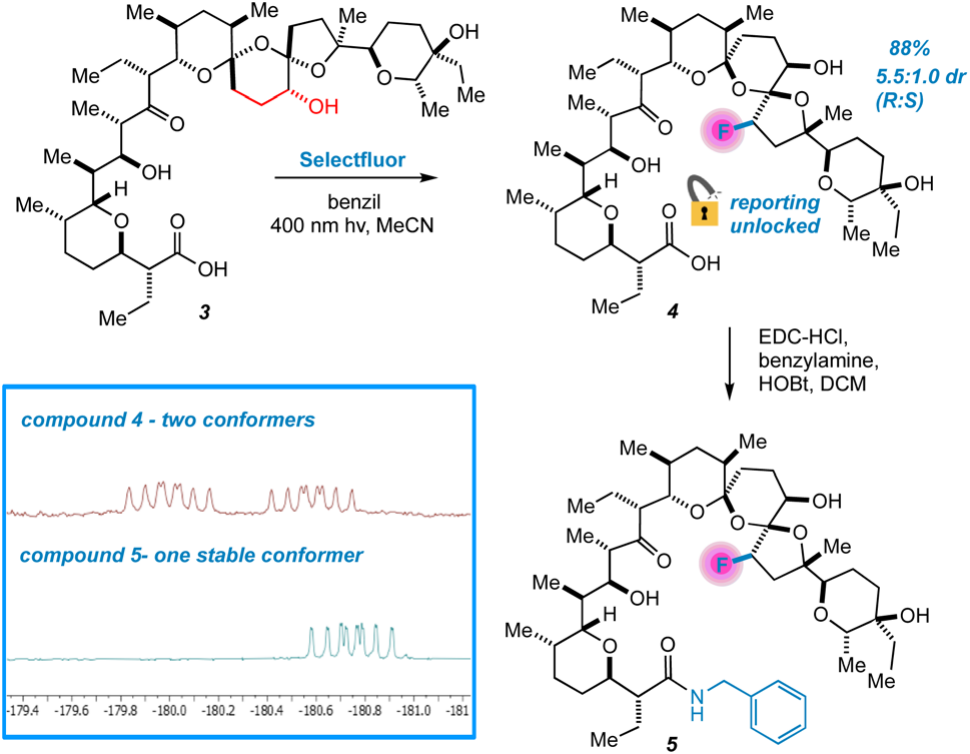
Selective fluorination of 3 unlocks conformational reporting from the SAL backbone.

Next, we changed tactics and hydrogenated the double bond of 1 to yield 18,19-dihydro SAL (3).^21^ We speculated that this would open additional sites that are unbound to metals using crystal structure guidance.^8,17^ To our great surprise, a highly regio- and diastereoselective reaction thereby takes place, accompanied by the appearance of two peaks in the ^19^F NMR spectrum of the crude reaction mixture (Fig. 4). We were initially uncertain as to the relationship between these two peaks. At first, we considered diastereomers, but their identical splitting patterns ruled this out. With a relative integration of 1.3 to 1.0, we then reasoned that the peaks could represent two conformers of similar thermodynamic stability separated by a significant barrier to interconversion.^22^ In this case, initial efforts at characterizing our compound would be convoluted by multiple conformers in solution. Fortunately, prior work presented by Huczyński and co-workers found that *N*-benzylamide derivatives of SAL adopt stabilized conformations through intramolecular H-bonding networks.^23^ Indeed, converting 4 to its corresponding *N*-benzylamide derivative prompted the ^19^F NMR peaks to coalesce with the additional benefit of facilitating purification by standard preparatory HPLC.

**Fig. 5:**
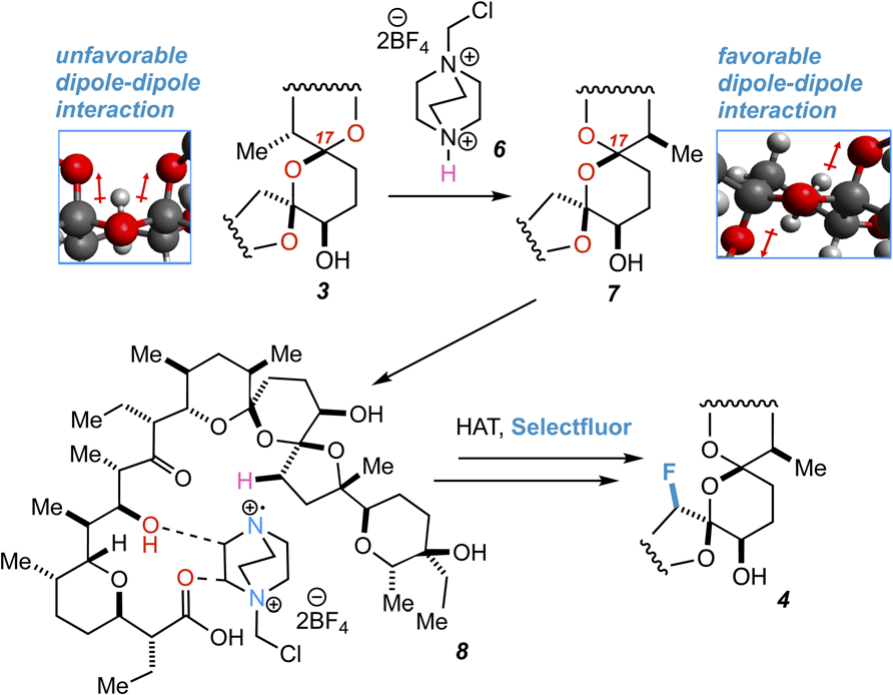
An origin for the selective fluorination of 3 is proposed.

Extensive characterization of the *N*-benzylamide of 4 led to the determination that the C22 position had been fluorinated with selectivity for the *R* isomer, an initially mysterious finding considering the scattershot fluorination of enone 2. However, further investigation also indicated epimerization of the C17 center (Fig. 5). HAT reactions initially generate organic acid catalyst 6, prompting rapid reconfiguration of the C17 center in 3 to epimer 7.

**Fig. 6:**
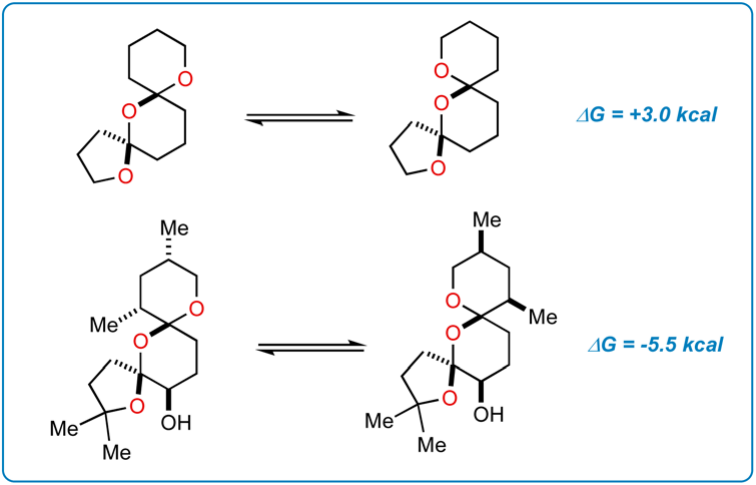
Stability of the core is dictated by substituents. Calculations done at (ωB97X-D/6-31+G*).

An origin for the prodigious selectivity observed in our reaction finally came into focus. It has previously been shown that epimerization of C17 in SAL allows for favorable dipole–dipole interactions about the bis-spiroketal ring system.^24^ The five-membered ring containing C22 is “flipped” to the interior of the SAL backbone in epimer 7, where carboxyl O1 and hydroxy O9 are able to direct SRD and Selectfluor through nonclassical hydrogen bonding interactions.^14^ The result is fluorinated product 4, which is deactivated to further epimerization events and degradation through inductive effects.^25^ Interestingly enough, simple density functional theory (DFT) calculations (ωB97X-D/6-31+G*) show that the epimerization is not driven by purely stereoelectronic considerations. In a stripped-down model system (Fig. 6), the “inverted” epimer is less stable, whereas in the more highly elaborated core, the inverted (C17) epimer is more stable. Enone 2, in contrast, is unable to epimerize and only experiences the initial nonselective HAT process, thus resulting in scattershot fluorination.

**Fig. 7:**
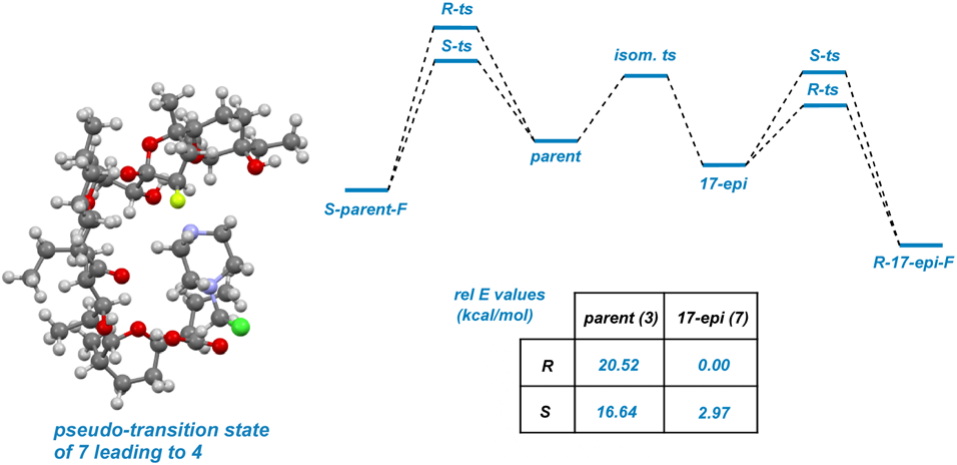
Pseudo-transition state leading to product 4; relative energies of pseudo-transition states.

**Fig. 8:**
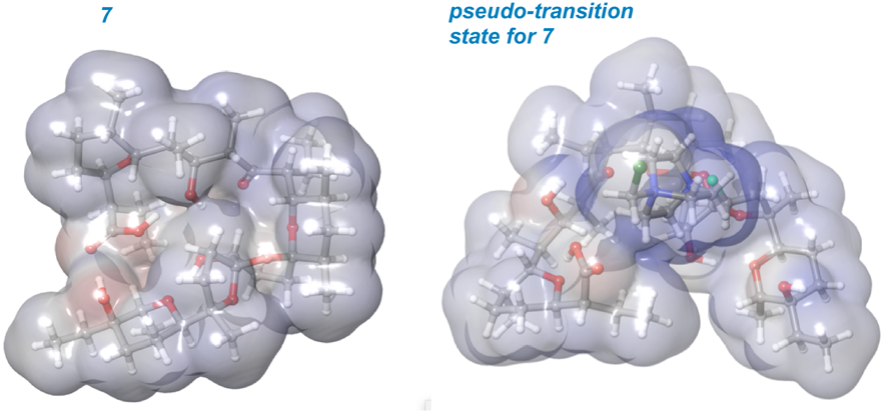
Electrostatic potential surfaces of 7 visualize cradling of charge in a pseudo-transition state.

To lend support to the mechanistic order of epimerization followed by fluorination, a series of molecular mechanics calculations were undertaken (Fig. 7). A molecule of SRD was tethered to either the *R* or *S* position on C22 in the fluorinated product arising from both parent compound 3 and epimerized backbone 7 to mimic the stereochemistry-determining stage of the reaction. The resulting energies can be interpreted as “pseudo-transition states” whereby epimerized backbone 7 demonstrates significantly favorable reactivity compared to parent compound 3. As illustrated in Fig. 7 and 8, epimerization permits the charged SRD species to dock along interior of the salinomycin backbone as a surrogate for a metal cation when reacting at C22. The pseudo-transition state leading to (*R*)-4 is found to have the lowest energy.

**Fig. 9:**
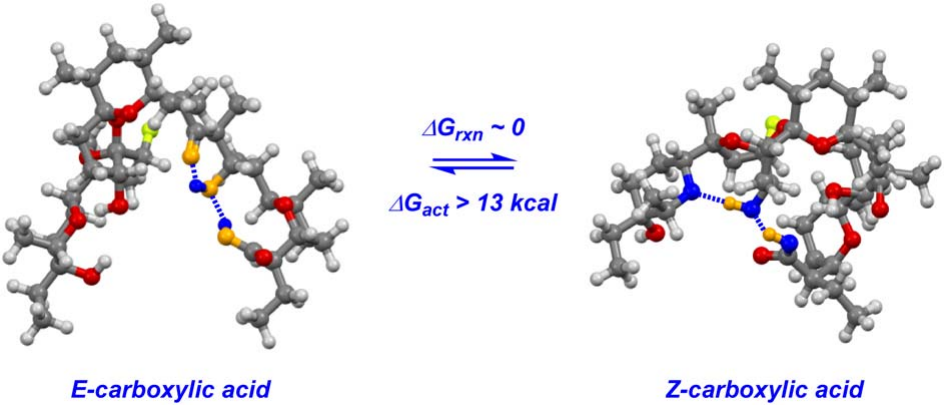
Carboxylic acid isomerization and reordering of hydrogen bonding networks leads to discrete conformations of 4.

**Fig. 10:**
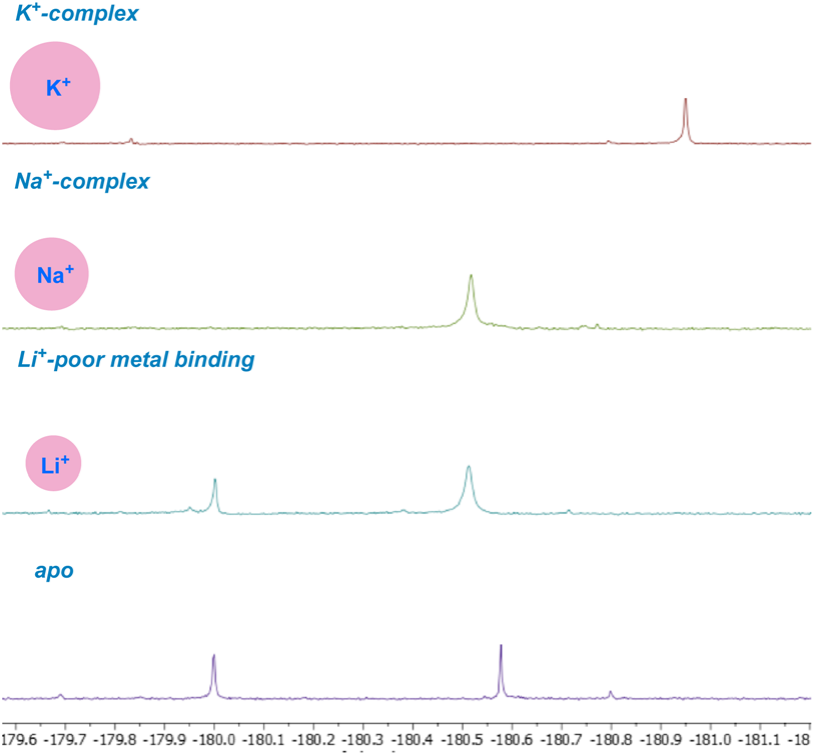
Metal-binding ability of fluorinated 4 screened by ^19^F NMR.

**Fig. 11:**
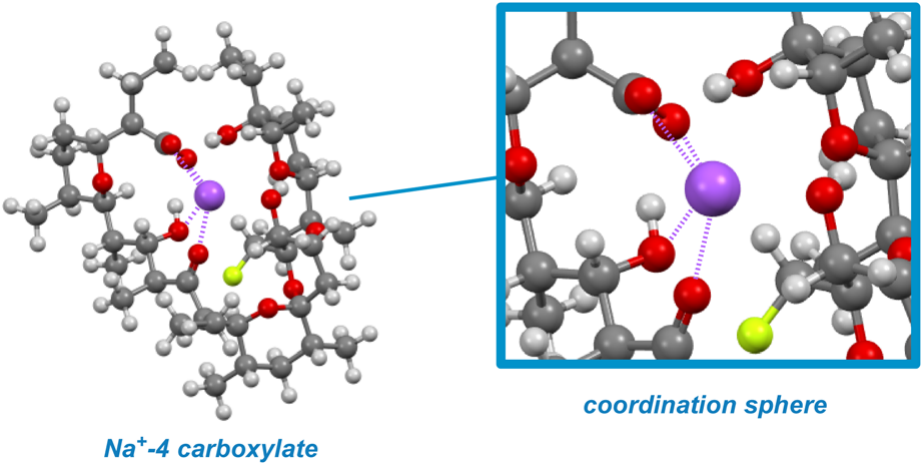
A Na^+^-4 carboxylate complex modelled using MCCS shows proximity of the fluorine nucleus to metal-binding substituents.

The key to understanding the two discrete conformers that interconvert slowly on the NMR time scale arises from different lines of evidence embedded in the coupled ^19^F NMR spectra. First, identical *J*-couplings measured for each conformer of 4 reveal that torsion in the bis-spiroketal system is not responsible for the two conformers.^26^ Additionally, only one conformer appears in the ^19^F NMR of the amide, implying that the carboxylic acid plays a key role in the energetic separation of the two conformers. This could be explained by *cis*–*trans* isomerization of the carboxylic acid,^27^ which possesses an expected barrier to interconversion of around 13 kcal (Fig. 9). Combine this with an associated disruption of the H-bonding networks of *E*- and *Z*-carboxylic acids leading to a further energetic penalty,^28^ and we have a viable explanation. In fact, MMFF-corrected force-field Monte Carlo calculations show that the two lowest energy conformers (virtually isoenergetic) have *E*- and *Z*-carboxylic acid geometries (Fig. 9). In each case, the calculated conformers comport with the *J*-couplings measured in the coupled ^19^F NMR spectra. In the case of amide 5, only one energetically viable conformer is predicted (the next is 5.3 kcal higher in energy), in line with our experimental observations. In this respect, our computational efforts were greatly aided by comparison of calculated geometries about the five-membered ring in 4 and 5 to anticipated torsional angles predicted by the ^19^F NMR data.^29^*The installed fluorine reporter thus provided a vital basis for the validation or rejection of each calculated structure*. The fluorine nucleus in product 4 is well-poised to be a conformational reporter in both a specific and general sense. It is positioned along the interior of the pseudo-macrocyclic backbone at a significantly strained and functionalized juncture. With a high sensitivity to chemical environment^30^ and anisotropic effects,^31^ we suspected that the fluorine nucleus in 4 could allow for the direct visualization of cation binding by ^19^F NMR (Fig. 10). In the presence of alkali metal salts and their corresponding carbonates, we find metal binding for 4 can be easily assessed. In keeping with previously determined binding affinities for parent SAL,^32^ we find that 4 has a great preference for binding K^+^ and Na^+^ relative to Li^+^. One can see that Li^+^ complexation offers little perturbation to the conformational equilibrium, whereas with Na^+^ and K^+^ binding the conformers merge into one metal-bound species in which the K^+^ complex ^19^F peak moves substantially upfield. In epimerized product 4, Monte Carlo conformational searches (MCCS) followed by optimization at DFT suggest that O21 is no longer a metal coordination site, and the installed fluorine nucleus is situated in close proximity to metal-binding substituents instead (Fig. 11).

## Conclusions

We have designed a reaction reliant on C–H bond activation to isomerize and fluorinate a widely studied bioactive skeletal backbone with high selectivity. In so doing, we find incorporation of a fluorine nucleus to enable monitoring of conformational dynamism by ^19^F NMR with high sensitivity and low-noise. In turn, this fluorine probe has been shown to elucidate an interesting structure–conformational relationship and has proven useful in determining metal-binding capability. We envisage that late stage C–H bond fluorination, when carefully engineered, can be exploited to simplify the study of complex, conformationally active molecules for a diverse range of applications. Further studies will focus on the precise metal binding characteristics of this unique system.

## Data availability

Experimental and computational data is located in the ESI.†

## Author contributions

J. Ruskin made initial discoveries, conducted selectivity screening, characterized products, and provided drafts of the manuscript. R. Dekeyser supplied computational findings supervised by T. Dudding. N. Garrison synthesized derivatives for screening. P. Williams and M. K.-Johansen assisted in selectivity screening. A. Majumdar assisted in product characterization. A. Huczyński provided analytically pure samples of salinomycin, academic consultation, and reviewed manuscript drafts. T. Lectka supervised the project, performed computations, and reviewed/edited the manuscript.

## Conflicts of interest

The authors declare no competing financial interest.

## Supplementary Material

SC-OLF-D5SC01857A-s001
