# Supplementary material for: A highly selective C–H bond fluorination unlocks conformational reporting in a complex natural product derivative
Source: Chem Sci. 2025 Mar 26;16(20):8729–34. doi: 10.1039/d5sc01857a (PMC12001048; doi:10.1039/d5sc01857a)
Supplement: SC-016-D5SC01857A-s001 [file SC-016-D5SC01857A-s001.pdf]

Supplementary Information (SI) for Chemical Science.
This journal is © The Royal Society of Chemistry 2025

Supporting Information

A Highly Selective C-H Bond Fluorination Unlocks Conformational Reporting in a Complex Natural Product Derivative

Jonah Ruskin,[a] Roxanne Dekeyser,[b] Nathaniel Garrison,[a] Phoebe Williams,[a] Maya Kramer-Johansen,[a] Ananya Majumdar,[a] Travis Dudding,*[b] Adam Huczyński*[c] and Thomas Lectka*[a]

[a]*Department of Chemistry, Johns Hopkins University, 3400 North Charles St., Baltimore, MD, 21218, United States*
[b]*Department of Chemistry, Brock University, St. Catharines, Ontario L2S3A1, Canada*
[c]*Department of Medical Chemistry, Faculty of Chemistry, Adam Mickiewicz University, Poznań, Poland*

Table of Contents

General Information..........S3

General Fluorination Procedure..........S3

Degradation of **1** in the Presence of Selectfluor..........S4

Salt-Free Fluorination of **2**..........S5

Deactivation of M^+-**2** complexes..........S6

Fluorination of **3**..........S7

Metal Complexation Screening..........S8

Syntheses of Starting Materials..........S8-S10

Conversion of **4** to *N*-Benzylamide **5**..........S10

Characterization Data for **5**..........S11

NMR Spectral Data..........S12-S20

Interpretation of NMR Data..........S21-S24

Mass Spectral Data for **5**..........S25

Computational Methods..........S26-S56

References..........S57-S58

General Information

Unless otherwise stated, all reactions were carried out under strictly anhydrous conditions and N_2 atmosphere. All solvents were dried and distilled by standard methods. All ^{1}H and ^{13}C NMR spectra were acquired on either an 800 MHz, 600 MHz NMR or 400 MHz NMR spectrometer in either CD_3CN, C_7D_8, or $CDCl_3$ as specified. All ^{19}F NMR spectra were acquired on a 400 MHz spectrometer or 300 MHz spectrometer in CD_3CN. The ^{1}H and ^{13}C NMR chemical shifts are given in parts per million (δ) with respect to an internal tetramethylsilane (TMS, δ = 0.00 ppm) standard. NMR data are reported in the following format: chemical shift (multiplicity (s = singlet, d = doublet, t = triplet, q = quartet, m = multiplet), coupling constants (Hz), integration). Spectral data were processed with MestreNova software. Photochemical reactions were run in front of a 72-LED work light (Designers Edge L1923) placed approximately six inches from the microwave vials without any filters. The microwave vials used were purchased from Chemglass products. HPLC purification was conducted on a Teledyne IscoCombiFlash EZ Prep system using a Dynamax-60A SiO2 column and HPLC grade EtOAc and hexanes. 4-Fluoro-3-nitrobenzotrifluoride was purchased from Tokyo Chemical Industry (prod no. F0324) and dissolved in CD_3CN, serving as an internal standard of known concentration for accurate ^{19}F NMR yield determination. Salinomycin sodium salt was isolated from Sacox® 120 micro Granulate which is an anticoccidial feed additive distributed by Huvepharma Polska. All other chemicals were used as purchased.

Salinomycin is a potent neurotoxin and should be handled with caution.

General Fluorination Procedure

To an oven-dried microwave vial equipped with a stir bar was added 0.067 mmol of SAL substrate (**1, 2** or **3**), Selectfluor (28 mg, 1.2 eq), and benzil (1 mg, 0.1 eq). The vial was then sealed with a cap with a septum using a crimper and evacuated/refilled with nitrogen gas three times. A degassed needle and syringe were used to transfer 1 mL of distilled acetonitrile to the microwave vial, and the reaction mixture was irradiated with a cool white LED work light while stirring. After 14 hours, a 0.1 mL aliquot was taken for ^{19}F NMR yield determination, and the reaction mixture was transferred to a separatory funnel, diluted with deionized water, and extracted into dichloromethane. The combined organic layers were washed with 15 mM H_2SO_4 followed by two additional portions of deionized water. Solvent was then removed under reduced pressure to yield the crude product.

*The photochemical conditions employed in this work have been previously developed.[1]

Degradation of 1 in the Presence of Selectfluor

To a microwave vial equipped with a stir bar was added 50 mg (1 eq, 0.067 mmol) of Salinomycin (**1**) and 28 mg (1.2 eq, 0.079 mmol) of Selectfluor. The vial was then sealed with a cap with a septum using a crimper and evacuated/refilled with nitrogen gas multiple times. A degassed needle and syringe were used to transfer 1 mL of anhydrous acetonitrile to the vial which was permitted to stir for 14 hours. An aliquot of the reaction mixture was taken, and degradation of the starting material was verified by ^{1}H NMR.

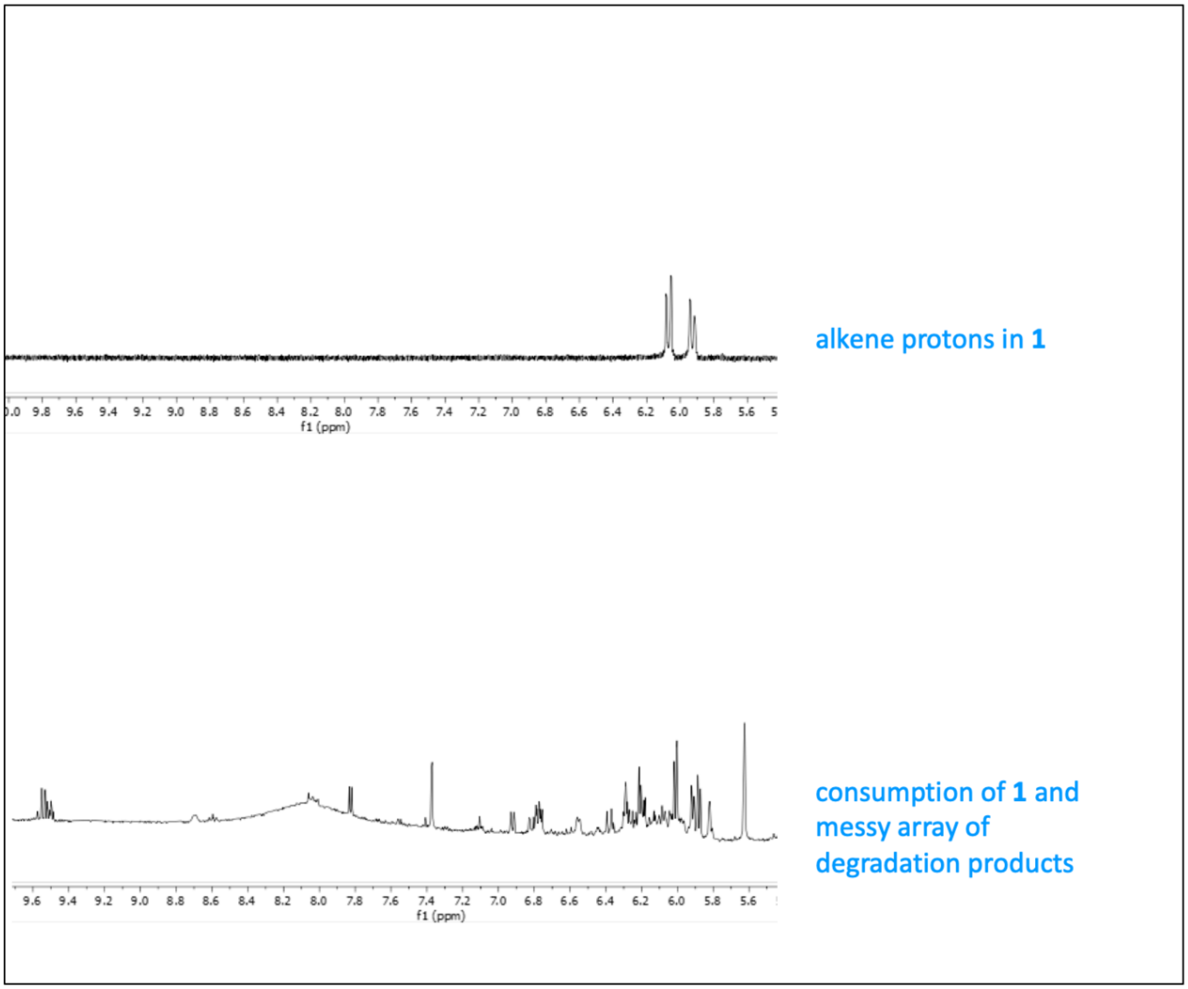

Figure S1. ^{1}H NMR spectra in CD_3CN of **1** (top) and degradation of **1** (bottom) after being stirred in the presence of Selectfluor.

Salt-Free Fluorination of 2

Compound **2** was first fluorinated in accordance with the general fluorination procedure.

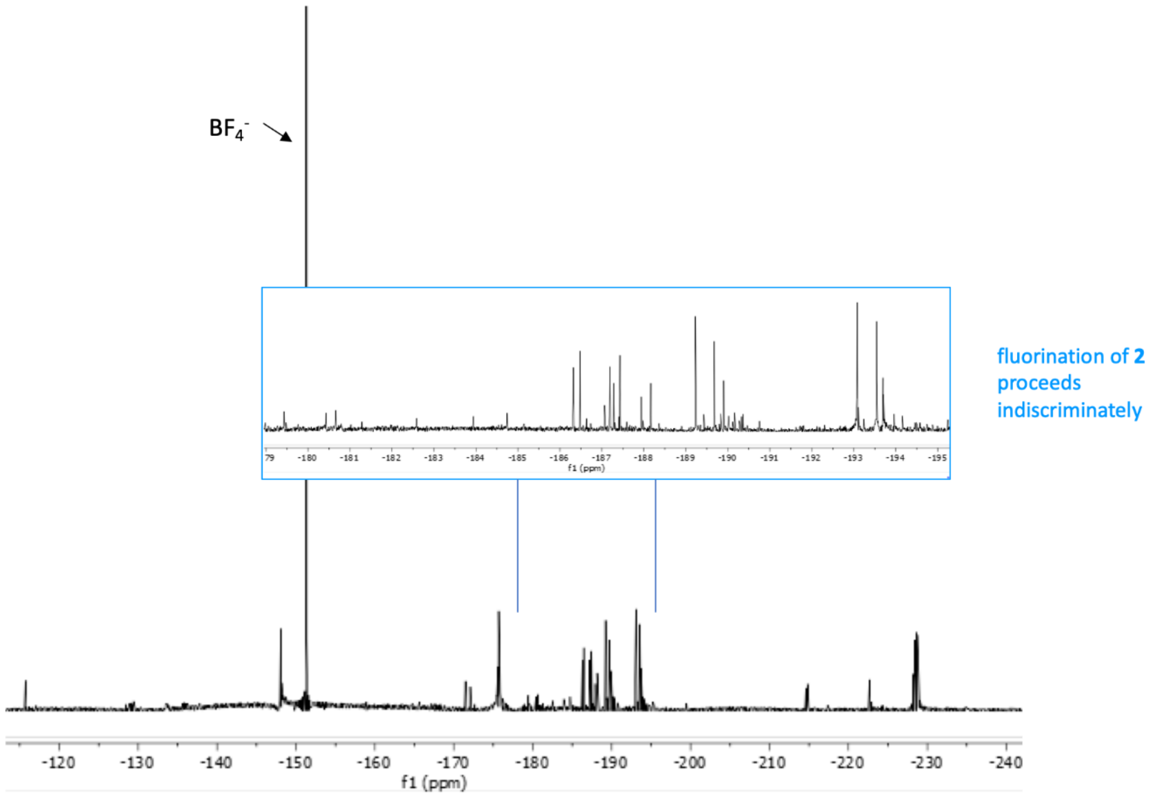

Figure S2. $^{19}F\{^{1}H\}$ NMR spectrum in CD_3CN of crude reaction mixture resulting from the fluorination of **2**.

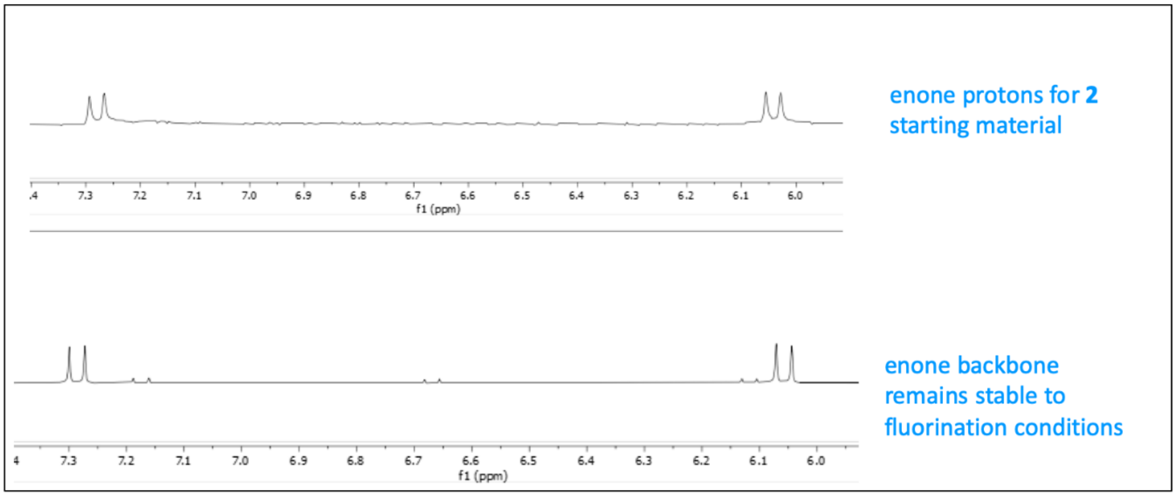

Figure S3. ^{1}H NMR spectra in CD_3CN highlighting enone protons for **2** in starting material (top) and crude fluorination reaction mixture (bottom) .

Deactivation of M^+-2 Complexes

To an oven-dried 5 mL pear shaped flask equipped with a stir bar was added 0.067 mmol of **2** and 1.2 equivalents of alkali metal salt (LiClO4, NaBF4, or KClO4). The flask was evacuated and refilled with N_2 gas several times. Anhydrous acetonitrile (1 mL) was then added to the pear-shaped flask, and the mixture was permitted to stir for an hour. Separately, Selectfluor (28 mg, 1.2 eq) and benzil (1 mg, 0.1 eq) were added to an oven-dried microwave vial equipped with a stir bar; the vial was then sealed with a cap with a septum using a crimper and evacuated/refilled with nitrogen gas multiple times. A degassed needle and syringe were used to transfer the SAL solution of **M^+-2** from the pear-shaped flask to the microwave vial, and the reaction mixture was irradiated with a cool white LED work light while stirring. After 14 hours, a 0.1 mL aliquot was taken for 19F NMR yield determination, and the rest of the reaction mixture was transferred to a separatory funnel, diluted with deionized water, and extracted into dichloromethane. The combined organic layers were washed with 15 mM H_2SO_4 followed by two additional portions of deionized water.

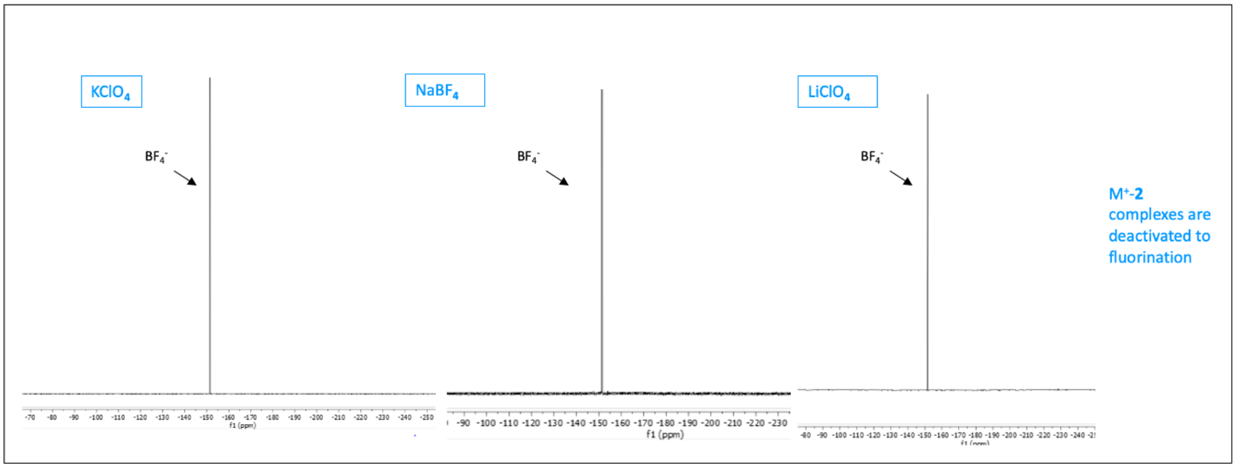

Figure S4. $^{19}F\{^1H\}$ NMR spectra in CD_3CN of crude reaction mixtures resulting from the fluorination of **2** in the presence of alkali metal salts.

Fluorination of 3

Compound **3** was first fluorinated in accordance with the general fluorination procedure.

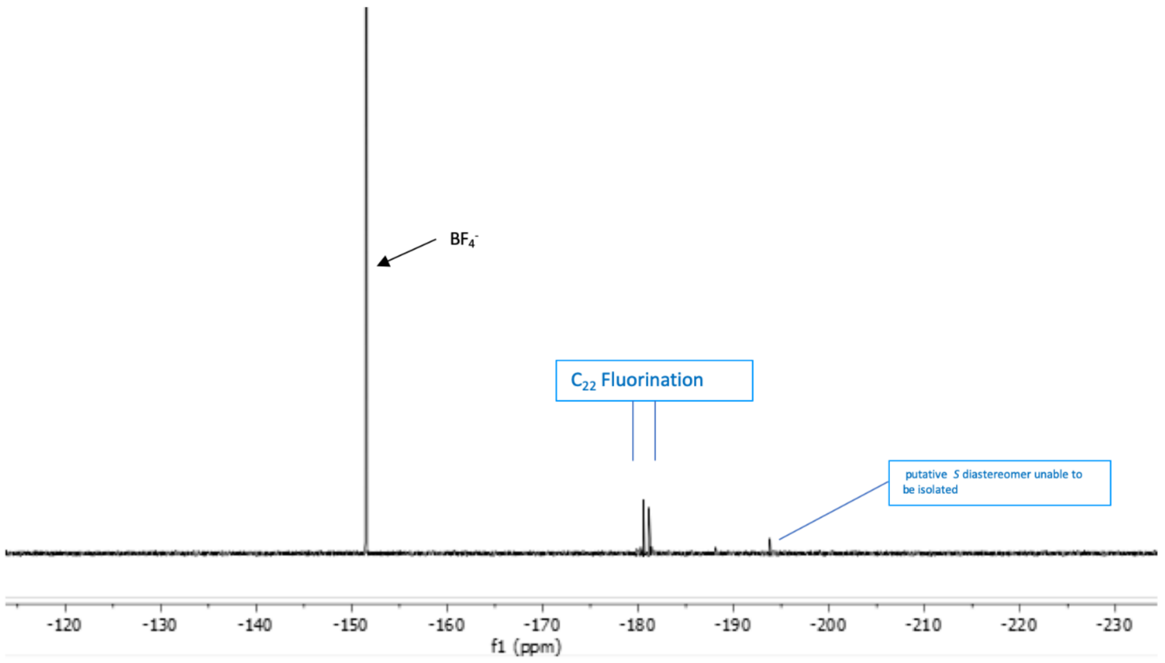

Figure S5. $^{19}F\{^1H\}$ NMR spectrum in CD_3CN of crude reaction mixtures resulting from the fluorination of **3**.

Metal Complexation Screening

Compound **3** was fluorinated in accordance with the general fluorination procedure. After workup, 5 milligrams of the reaction mixture (0.006 mmol of **4)** was transferred to an oven-dried microwave vial equipped with a stir bar. To this was added 3 eq. of alkali metal salt ($LiClO_4$, $NaBF_4$, or $KClO_4$) and 3 eq. of corresponding base (Li_2CO_3, $NaHCO_3$, or $KHCO_3$). The vial was then sealed with a cap with a septum using a crimper and evacuated/refilled with nitrogen gas three times. A degassed needle was then used to transfer 0.5 mL of CD_3CN to the microwave vial. After permitting to stir for 12 hours, the mixture was transferred to an NMR tube for analysis by ^{19}F NMR. Chemical shifts are reported in reference to 4-Fluoro-3-nitrobenzotrifluoride.

Syntheses of Starting Materials

Isolation of salinomycin (**1**)

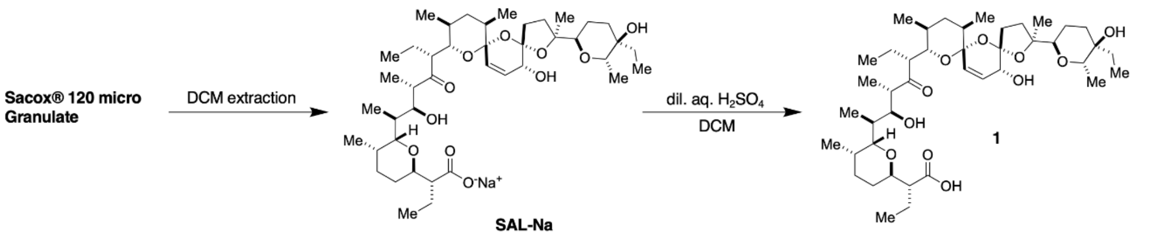

Salinomycin sodium salt (**SAL-Na)** was isolated from from Sacox® 120 micro Granulate, a veterinary feed additive that contains 12% **SAL-Na**. Material from a 100 g portion of Sacox® mixture was dissolved in dichloromethane, and crude salinomycin sodium salt was then purified by dry column vacuum chromatography. The spectroscopic data of obtained **SAL-Na** were in agreement with previously published literature. The purified **SAL-Na** was then dissolved in dichloromethane and stirred vigorously with a layer of aqueous sulfuric acid (pH = 1.5). The organic layer containing **1** was washed with distilled water, and then dichloromethane was evaporated under reduced pressure to dryness giving 6 g of **1**. The spectroscopic data of **1** were in agreement with previously published assignments.[2]

Synthesis of **2**

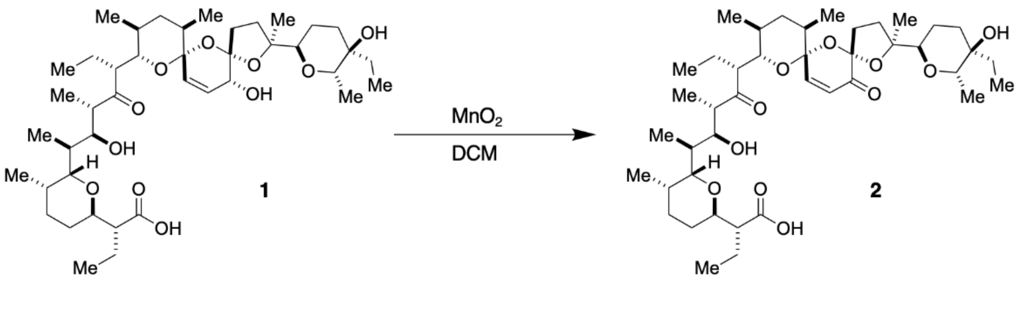

To a 100 mL round bottom flask equipped with a stir bar was added 500 mg of salinomycin (**1**) (0.667 mmol, 1 eq) which was dissolved in 40 mL of dry dichloromethane. To this solution was added 2.315 g (26.67 mmol, 40 eq) of MnO_2 in a single portion. The reaction mixture was permitted to stir for 18 hours before being diluted with dichloromethane and filtered through a pad of celite. Crude **2** was then purified on silica eluting with a gradient of 0-100% EtOAc/hexanes. The collected fractions were concentrated under reduced pressure, dissolved in dichloromethane and washed with 15 mM H_2SO_4 in a separatory funnel. Dichloromethane was then removed under reduced pressure to yield 250 mg of **2** (50%) as a white solid. Spectral data matched existing literature.[3]

Synthesis of ***3***

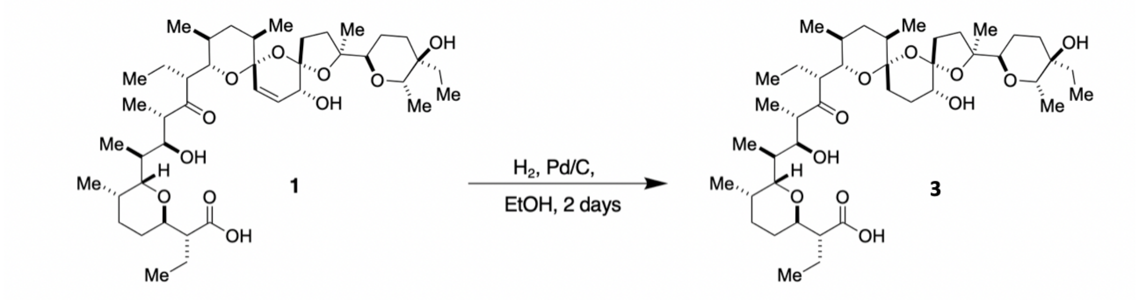

To a 50 mL round bottom equipped with a stir bar was added 300 mg of salinomycin (**1**) which was dissolved in 15 mL of absolute ethanol. To this solution was added 30 mg of 10% Pd/C in a single portion. A hydrogen atmosphere was then created using a balloon, and the reaction mixture was stirred overnight. The hydrogen balloon was refilled after 24 hours, and the reaction mixture was stirred for an additional 24 hours before being diluted with more ethanol and filtered through a pad of celite. Crude **3** was then purified on silica eluting with a gradient of 0-100% EtOAc/hexanes. The collected fractions were concentrated under reduced pressure,

dissolved in dichloromethane and washed with 15 mM H_2SO_4 in a separatory funnel. Dichloromethane was then removed under reduced pressure to yield 270 mg of **3** (90%) as a white solid. Spectral data matched previous literature.[4]

Conversion of 4 to *N*-Benzylamide 5

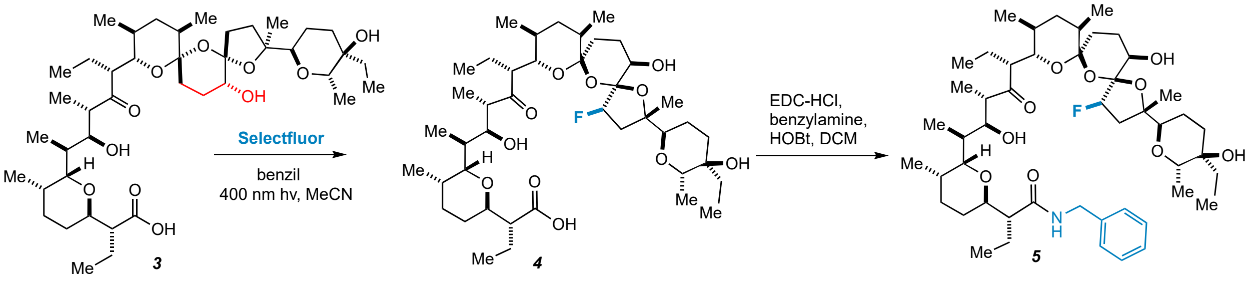

Compound **3** (50 mg, 0.067 mmol, 1 eq) was fluorinated in accordance with the general fluorination procedure to yield **4**. After workup, the crude reaction mixture was dissolved in 2 mL of DCM, transferred to a 5 mL round bottom flask equipped with a stir bar, and cooled in a salted ice bath. To this was added EDC-HCl (19 mg, 0.101 mmol, 1.5 eq) followed by 1-Hydroxybenzotriazole Monohydrate (5 mg, 0.034 mmol, 0.5 eq). The reaction mixture was kept at 0°C for four hours before being allowed to warm to room temperature. After 24 hours, the reaction mixture was diluted with DCM and washed with deionized water. Solvent was removed under reduced pressure to yield a yellow oil. The crude reaction mix was then taken up in EtOAc and passed through a short silica plug eluting with 100% EtOAc. The crude reaction mixture was then purified by normal phase preparative HPLC. One fraction contained analytically pure **5** yielding 12 mg of clear oil.

Characterization Data for 5

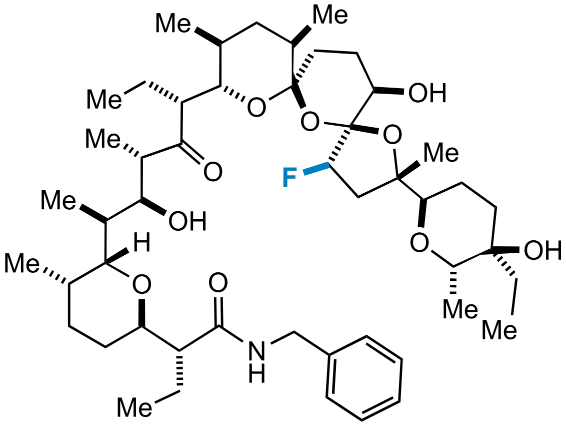

Compound **5**. Prepared as detailed on page S-10.

^{1}H NMR (CD_3CN, 600 MHz): δ = 7.35-7.29 (m, 4H), 7.24 (t, *J*= 7.4, 1H), 7.09 (t, *J* = 5.6 Hz, 1H), 4.93 (s, 1H), 4.82 (dd, *J* = 53.7, 4.2 Hz, 1H), 4.42 (m, 2H), 4.16 (ddd, *J* = 8.4, 6.2, 2.8 Hz, 1H), 4.02 (m, 1H), 3.97 (d, *J*= 5.6 Hz, 1H), 3.87-3.81 (m, 2H), 3.69 (q, *J* = 7.9 Hz, 1H), 3.63 (dd, *J* = 10.0, 2.4 Hz, 1H), 3.53 (m, 1H), 2.96 (m, 1H), 2.90 (ddd, *J* = 7.8, 4.9, 2.4 Hz, 1H), 2.83 (td, *J* = 11.2, 4.5 Hz, 1H), 2.74 (s, 1H), 2.39 (ddd, *J* = 45.4, 14.8, 4.2 Hz, 1H), 2.02 (m, 1H), 1.88-1.80 (m, 3H), 1.79-1.71 (m, 4H), 1.66 (m, 1H), 1.61-1.56 (m, 4 H), 1.49 (m, 1H), 1.45-1.40 (m, 4H), 1.30-1.27 (m, 5H), 1.27-1.17 (m, 6H), 1.05 (m, 1H), 0.93 (d, *J* = 6.9 Hz, 3H), 0.89-0.86 (m, 6H), 0.85-0.82 (m, 9H), 0.79 (d, *J* = 6.5 Hz, 3H), 0.73 (d, *J* = 6.9 Hz, 3H).

^{13}C {^{1}H} NMR (CD_3CN, 150 MHz): δ = 217.1, 176.5, 140.7, 129.3, 128.7, 127.8, 110.1, 106.2 (d, *J* = 21.8 Hz), 98.2 (d, *J* = 182.7 Hz), 86.8, 81.4, 78.1, 76.5, 76.1, 75.8, 72.1, 71.4, 71.2, 55.8, 50.4, 47.9, 43.7, 41.4 (d, *J* = 19.5 Hz), 38.8, 38.2, 37.4, 36.3, 33.6, 31.8, 30.2, 29.0, 26.9, 25.3, 23.4, 22.9, 22.0, 20.8, 19.5, 18.0, 16.5, 15.3, 14.1, 14.0, 12.2, 11.5, 7.6, 6.8.

^{19}F NMR (CD_3CN, 376 MHz): δ = -180.66 (dddd, *J* = -53.7, 45.4, 24.6, 2.8 Hz)

HRMS (ESI-FTICR-MS) m/z $C_{49}H_{79}FNO_{10}$: $[M + H]^+$ calcd. 860.5688, found 860.5666

NMR Spectral Data

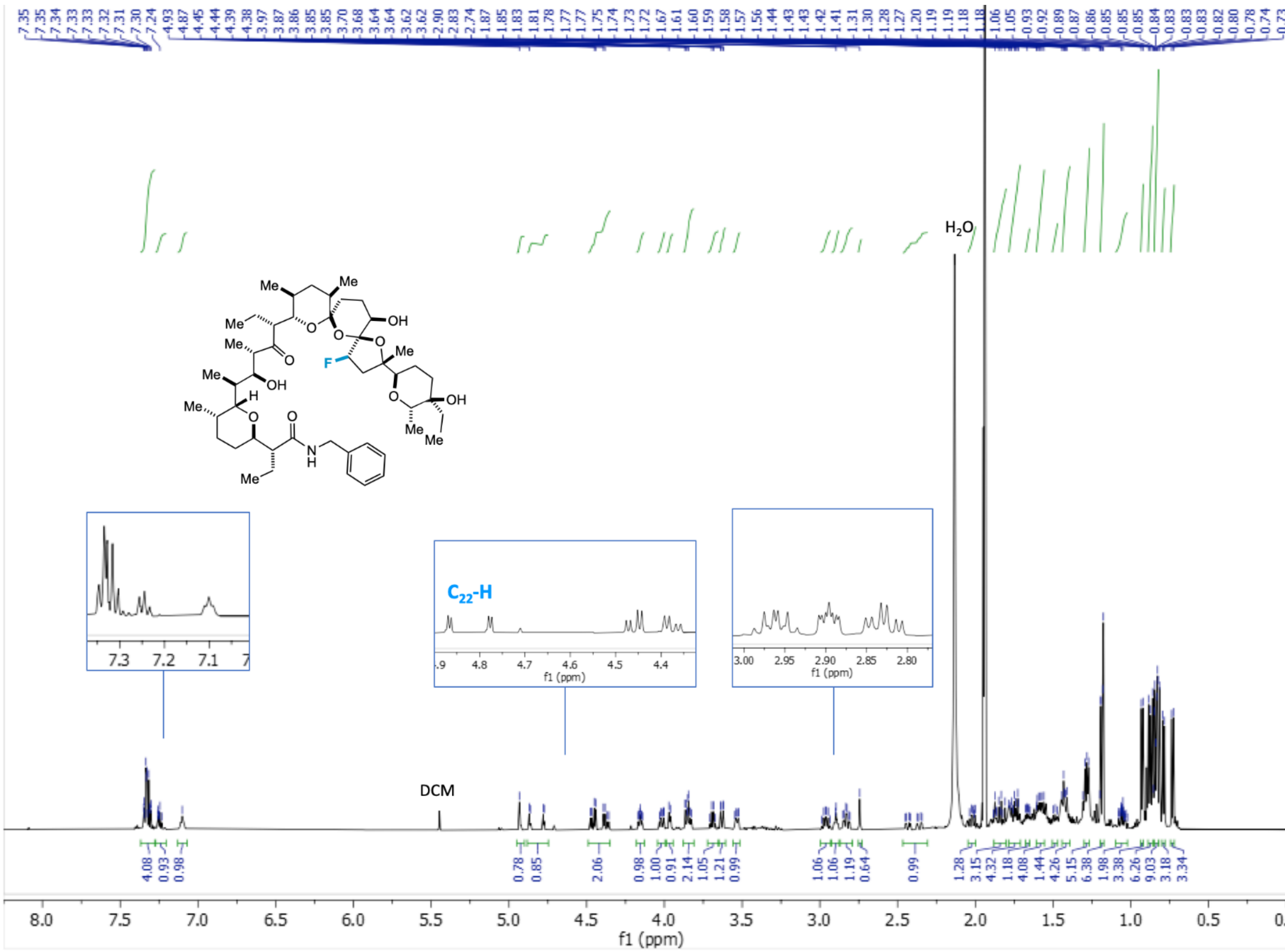

Figure S6. ^{1}H NMR (CD_3CN, 600 MHz) spectrum of compound **5**.

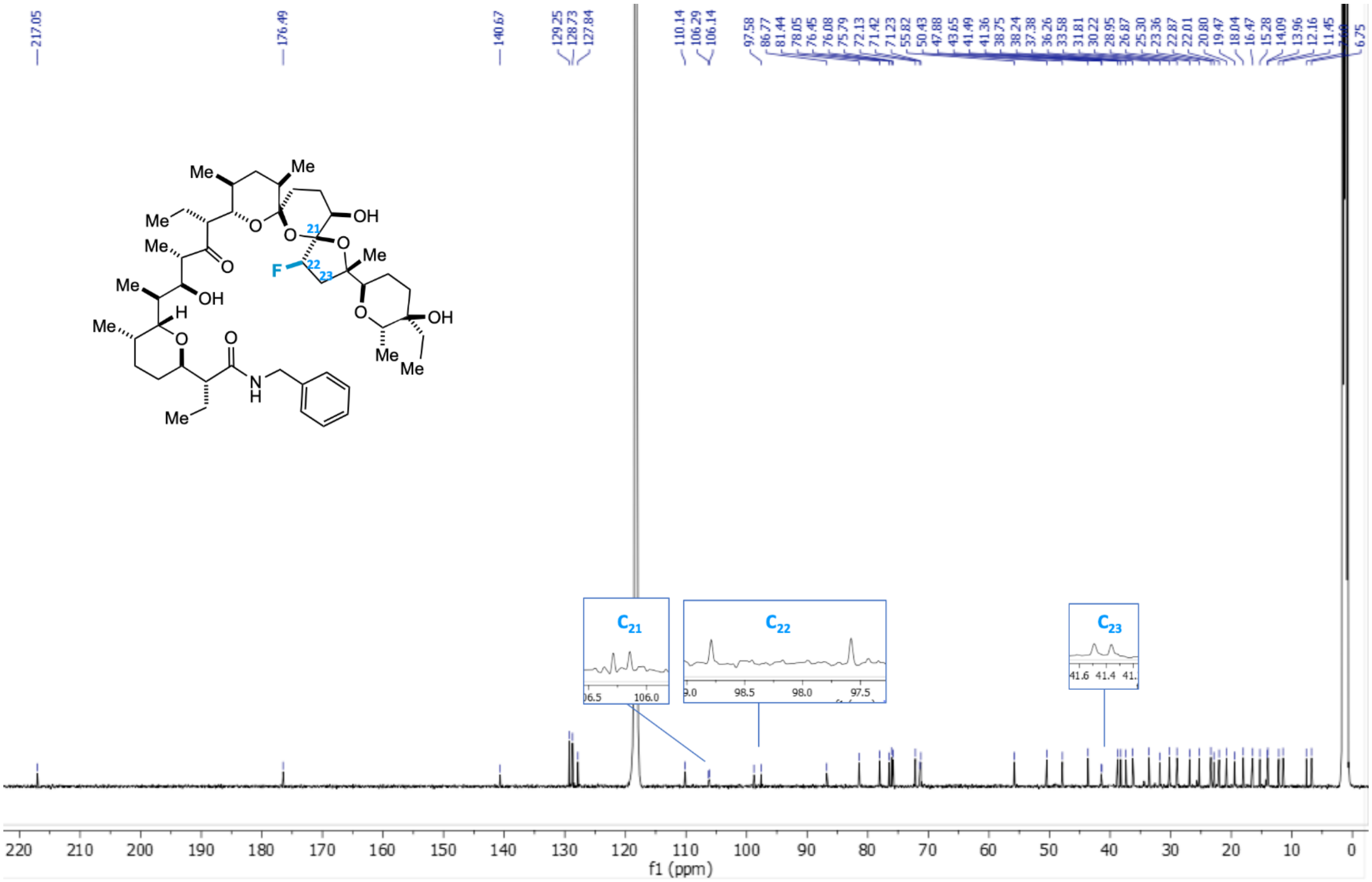

Figure S7. ^{13}C NMR (CD_3CN, 150 MHz) spectrum of compound **5**.

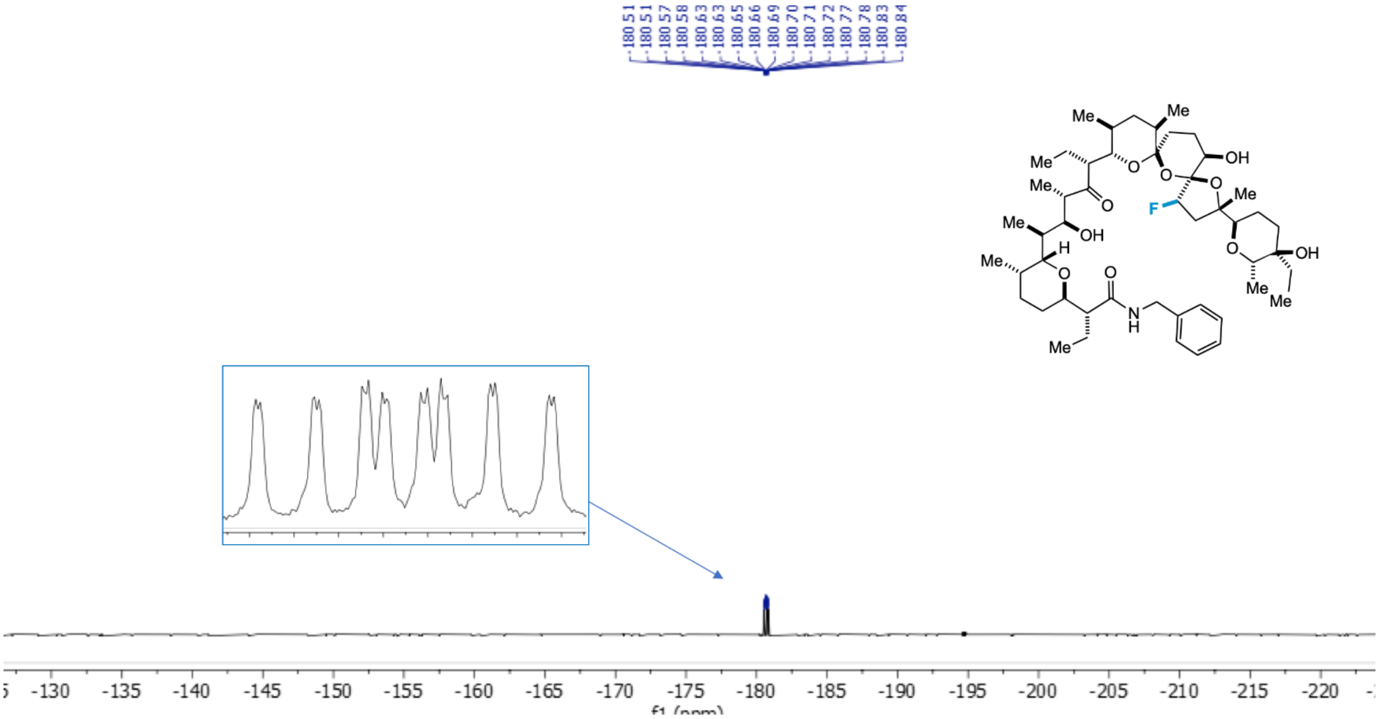

Figure S8. ^{19}F NMR (CD_3CN, 376 MHz) spectrum of compound **5**.

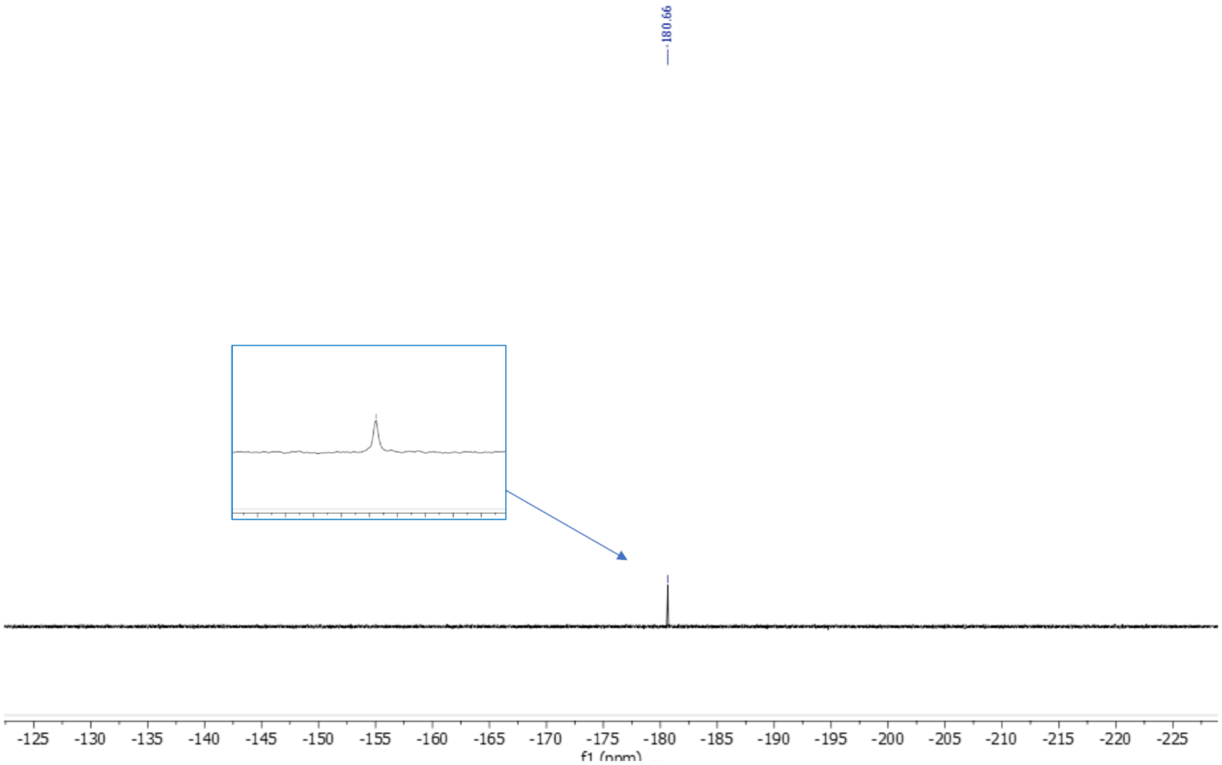

Figure S9. $^{19}F\{^1H\}$NMR (CD_3CN, 376 MHz) spectrum of compound **5**.

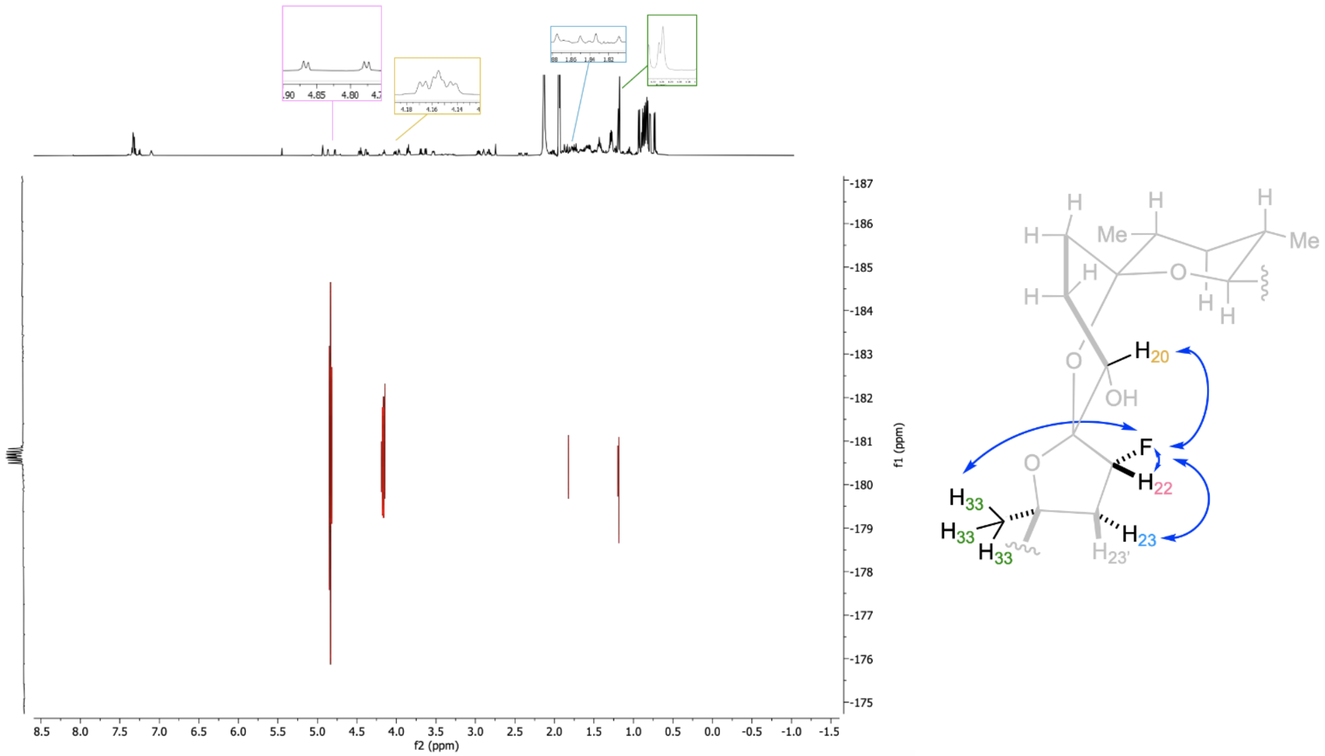

Figure S10. A ^{19}F-^{1}H Heteronuclear Overhauser Effect Spectroscopy (HOESY) experiment of compound **5** in CD_3CN is shown. The experiment was conducted on a 300 MHz instrument. 1D spectra collected on higher magnetic field instruments is placed on either axis.

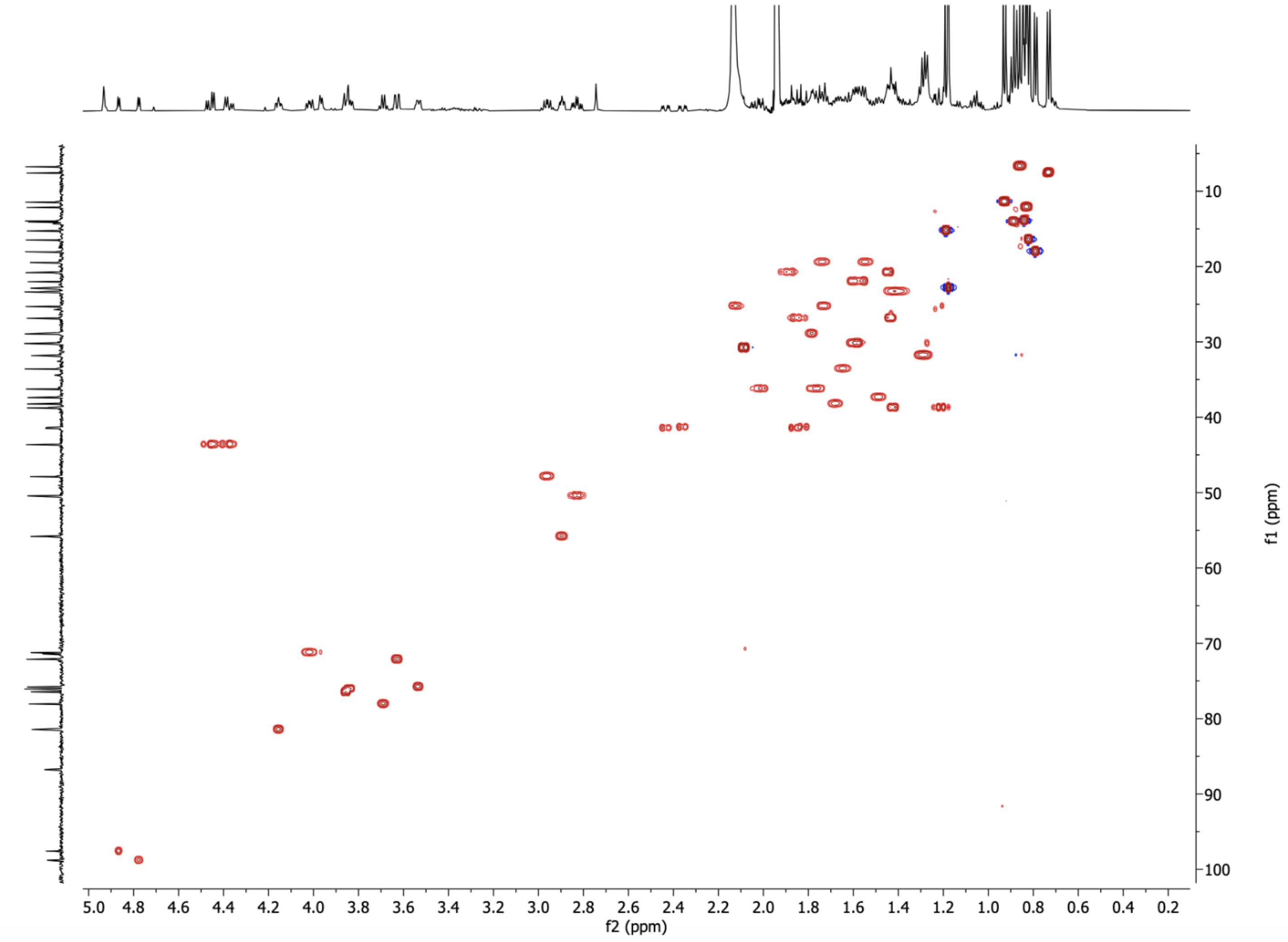

Figure S11. A 2D ^{1}H-$^{13}C\{1H\}$ Heteronuclear single quantum coherence (HSQC) experiment, without 19F decoupling along either axis is shown for compound **5** in CD_3CN. The experiment was conducted on a 600 MHz NMR spectrometer.

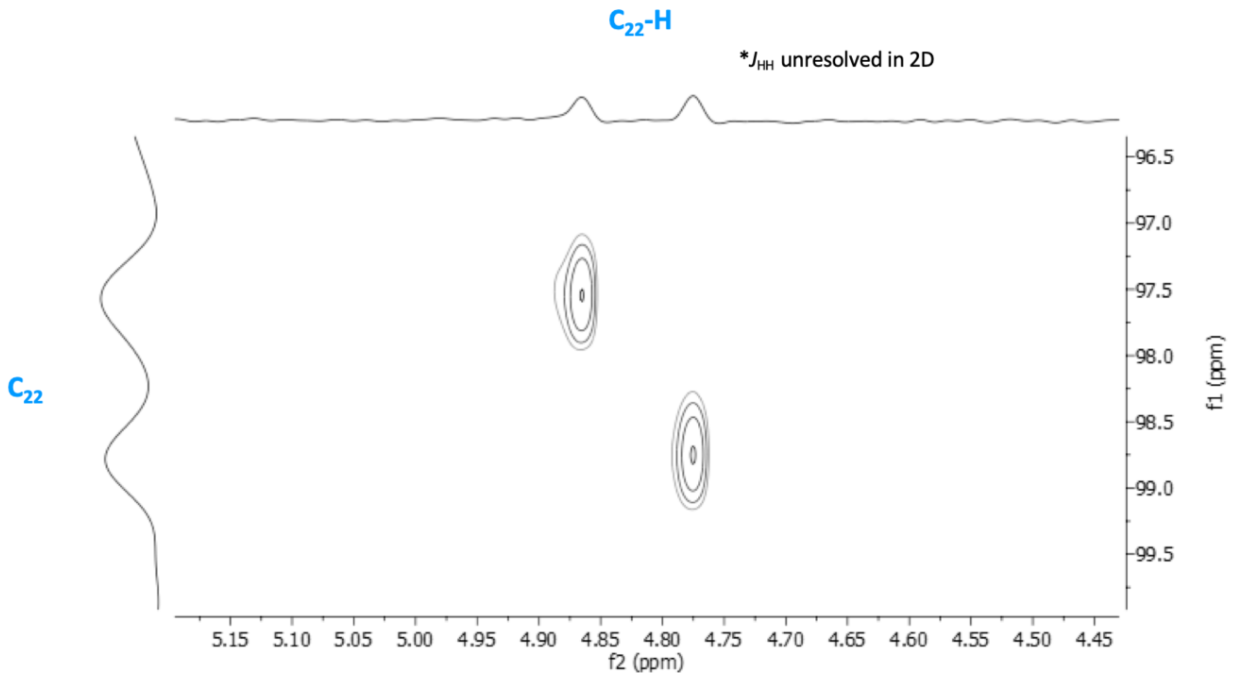

Figure S12. A 2D ^{1}H-$^{13}C\{1H\}$ Heteronuclear single quantum coherence (HSQC) experiment, without 19F decoupling along either axis is shown for the fluorine-coupled peaks in the ^{1}H NMR and $^{13}C\{1H\}$ NMR spectra of compound **5** in CD_3CN. The experiment was conducted on a 600 MHz NMR spectrometer.

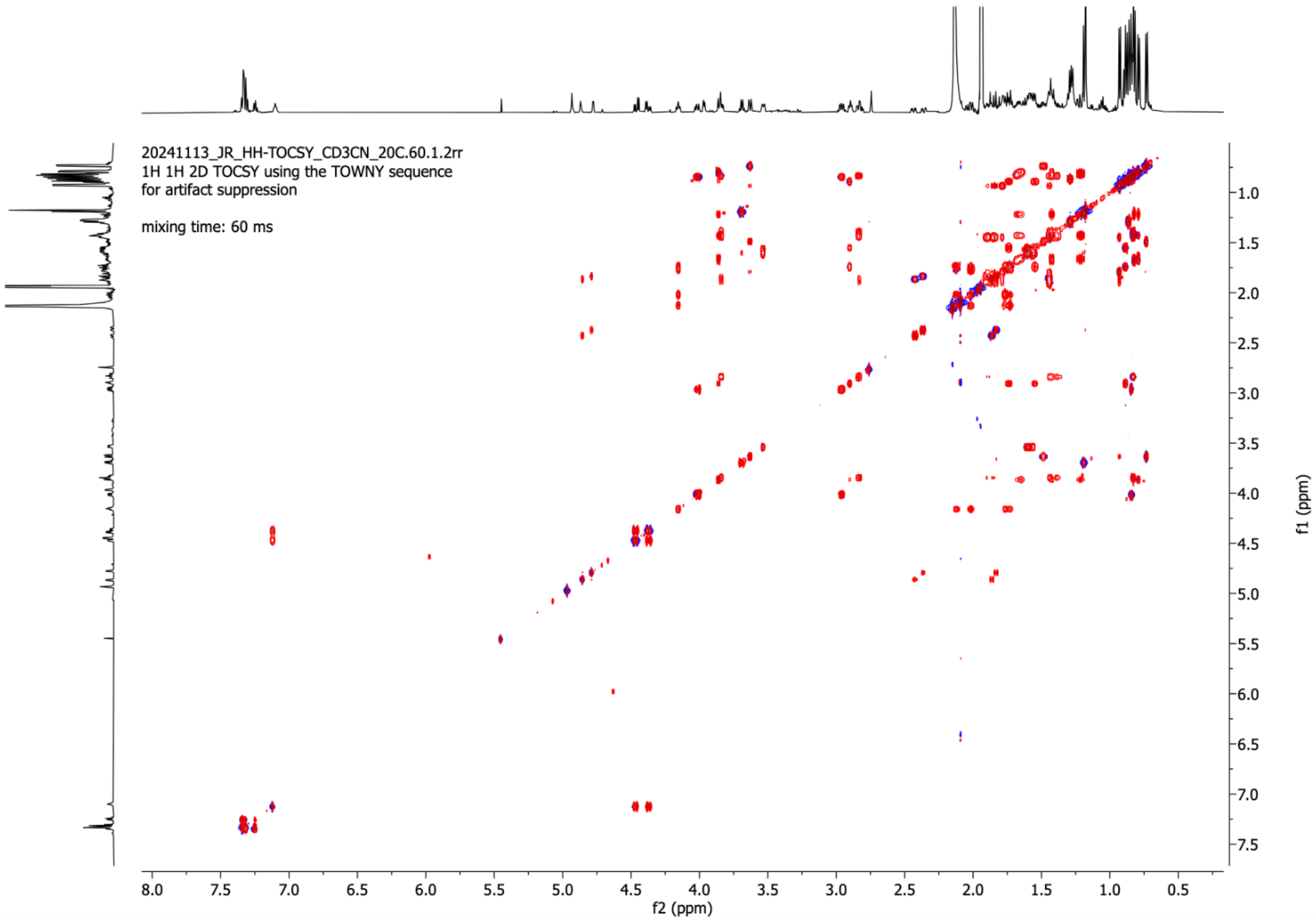

Figure S13. A 2D ^{1}H-^{1}H Total Correlation Spectroscopy (TOCSY) experiment is shown for compound **5** in CD_3CN. The experiment was conducted using an 800 MHz instrument.

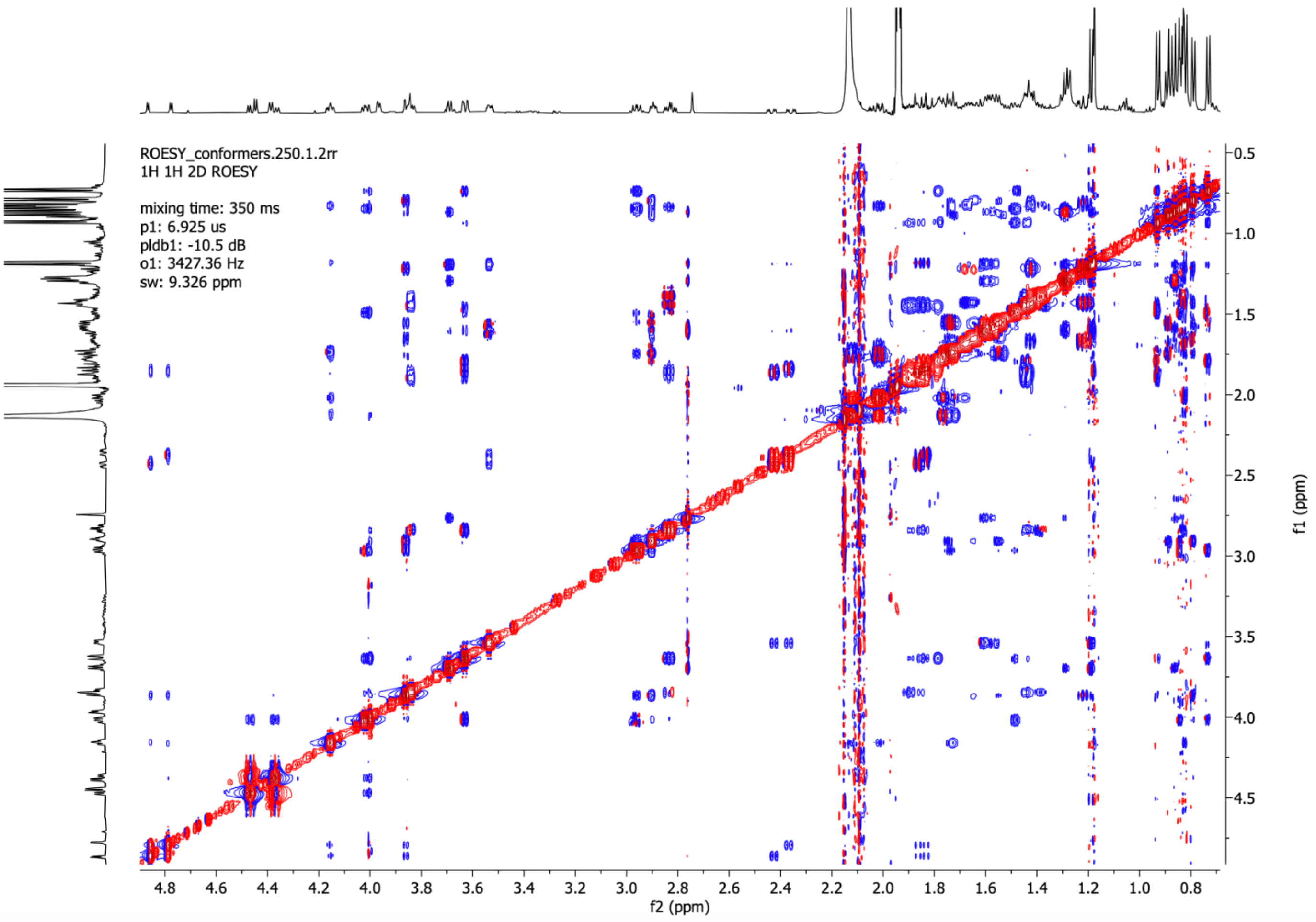

Figure S14. A 2D ^{1}H-^{1}H Rotating Frame Overhauser Enhancement Spectroscopy (ROESY) experiment with a mixing time of 350 ms is shown for compound **5** in CD_3CN. The experiment was conducted on an 800 MHz instrument.

Interpretation of NMR Data

Compound **5** was characterized by 1D ^{19}F NMR, 1D ^{1}H NMR, and 1D ^{13}C NMR along with the following 2D experiments:
^{1}H-^{13}C HSQC
^{1}H-^{13}C LR-HSQC
^{1}H-^{1}H TOCSY
^{1}H-^{1}H ROESY
^{19}F-^{1}H HOESY

All experiments were collected in CD_3CN and replicated in toluene-d_8.

Site of Fluorination

The ^{19}F NMR peak for **5** has a chemical shift (-181.66 ppm) consistent with fluorination at a secondary site. Additionally, one of the $^{2}J_{CF}$ couplings in the 1D ^{13}C NMR spectrum of **5** is to a quaternary center in the bis-spiroketal system. In the absence of 2D NMR data, this can only be explained by fluorination at either C18 or C22. The 2D ^{1}H-^{1}H TOCSY experiment reveals that the proton geminal to the fluorine nucleus is in a spin system with just two other protons, clarifying that the C22 position has been fluorinated on the five membered ring. The 2D ^{19}F-^{1}H HOESY experiment results in a clear NOE correlation between the installed fluorine and the C33 methyl group, placing the two substituents on the same face of the five-membered ring. This is corroborated by a NOE correlation in the 2D ^{19}F-^{1}H HOESY between the fluorine nucleus and one of the C23 protons. This C23 proton also has a strong ROE correlation with the C33 methyl group in the ^{1}H-^{1}H ROESY experiment. Therefore, product **5** has a fluorine substituent at the C22 position with *R* stereochemistry.

Epimerization

Our first indication that fluorinated product **5** has an epimerized C17 center relative to parent compound **3** came from an unusual ROE correlation between C13-H and C22-H in the ^{1}H-^{1}H ROESY experiment. The seemingly anomalous correlation is present using a variety of mixing times (100 ms, 175 ms, 350 ms) in both CD_3CN and toluene-d_8. This interaction could not be accounted for by the parent configuration of **3**. We immediately began to suspect that isomerization had occurred during the course of our fluorination.

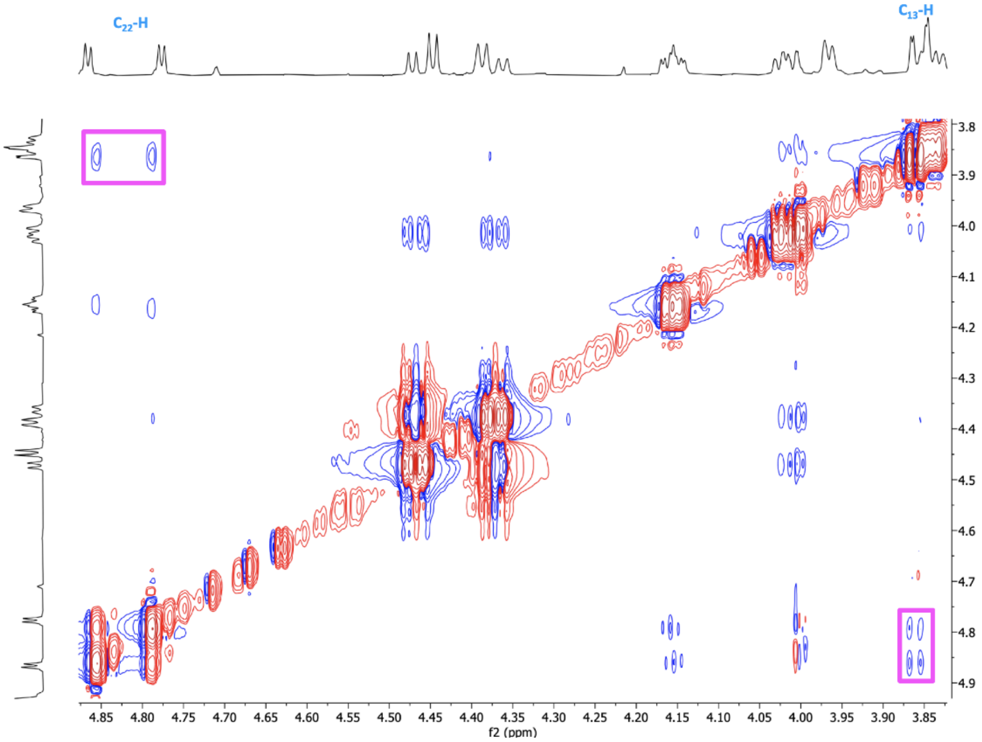

Figure S15. An unexpected correlation in the ^1H-^1H ROESY experiment for **5** indicates that epimerization has occurred.

Fortunately, Leadlay and co-workers reported extensive NMR characterization data for bioengineered SAL derivatives featuring C(sp^3) hybridized C18 and C19 positions.[5] The two bioengineered products they report are substituted with a hydroxy group at the C19 position and differ only by epimerization about the C17 quaternary center.[5] Comparison of our experimental findings to their data corroborated that C17 epimerization had occurred.

Both C22 substituents for compound **5** have a ROE correlation with C20-H, indicating that they are on the same face of the C ring. Thus, **5** has the same C21 configuration as parent compound **3**.[5] Findings thereafter further supported that C17 had epimerized. For the parent configuration observed in **3**, for example, one would expect a strong ROE correlation between one of the C18-H protons and C13-H and C15-H on the B ring. Instead, there is only a ROE correlation between C18-H and C16-H in isolated **5**, providing another match to the literature.[5]

Leadlay and co-workers also find that the C ring adopts a twist-boat conformation in their C17 epimerized product while the C ring adopts a chair conformation for their product with native configuration.[5] Initially, overlap of peaks in the ^1H NMR spectrum of **5** in CD_3CN prevented us from measuring *J*-couplings for either of the C18-H or C19-H peaks. This problem was solved by repeating our NMR experiments in deuterated toluene, where one of the C19-H peaks was fully resolved. A series of 8.0 to 8.4 Hz ^3J- coupling constants measured for C19-H indicated that the C ring in **5** is a twist boat.[5]

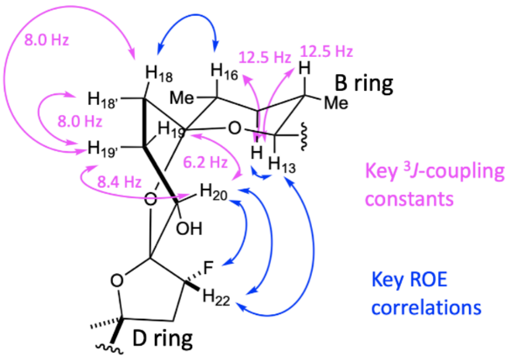

Figure S16. Key ROE correlations and ^3J-coupling values about the bis-spiroketal ring system are shown for **5**.

With the C ring in a twist boat conformation, Monte Carlo conformational searches on free acid **4** clearly predict the ROE correlation between C13-H and C22-H. As a note, calculated conformers could easily be validated or rejected based on torsional angles observed in the five-membered D ring.[6] Measuring the coupling constants in the 1D ^{19}F NMR spectrum of **5**, an anticipated orientation of substituents in the D ring could be drafted.[6]

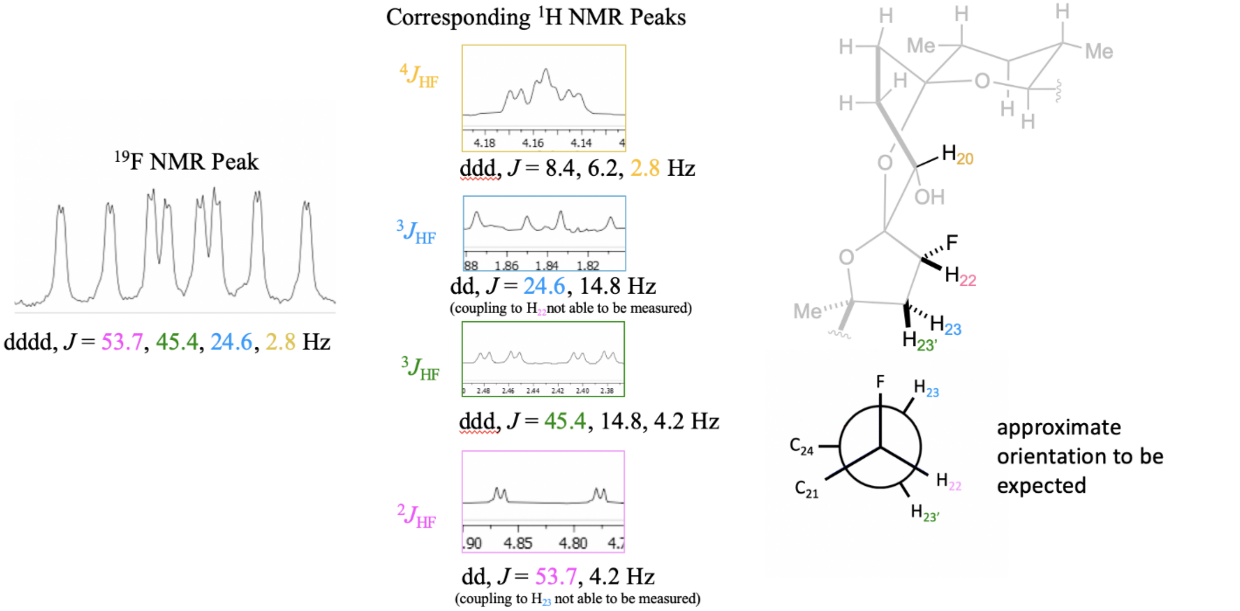

Figure S17. J-coupling values embedded in the ^{19}F NMR spectrum of **5** help anticipate torsional angles in the D ring.

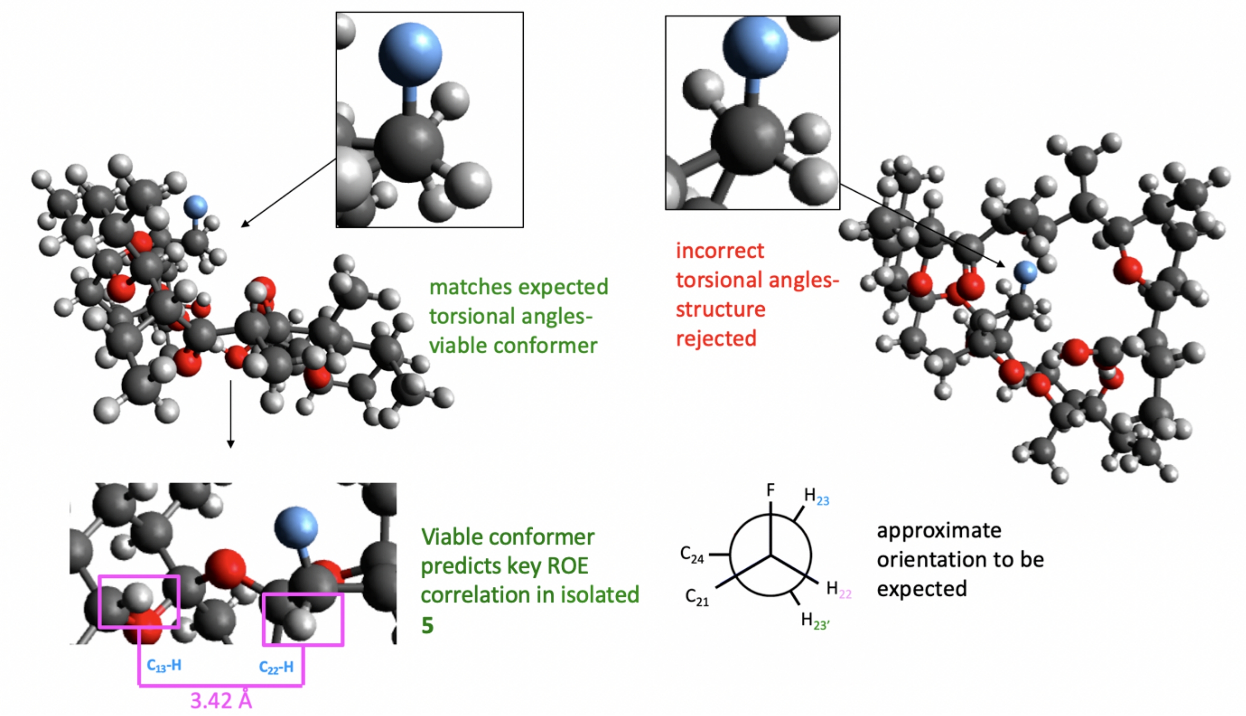

Figure S18. Predicted torsional angles in the D ring aid in screening computational results. Viable computed conformers also predict a key ROE correlation.

Mass Spectral Data

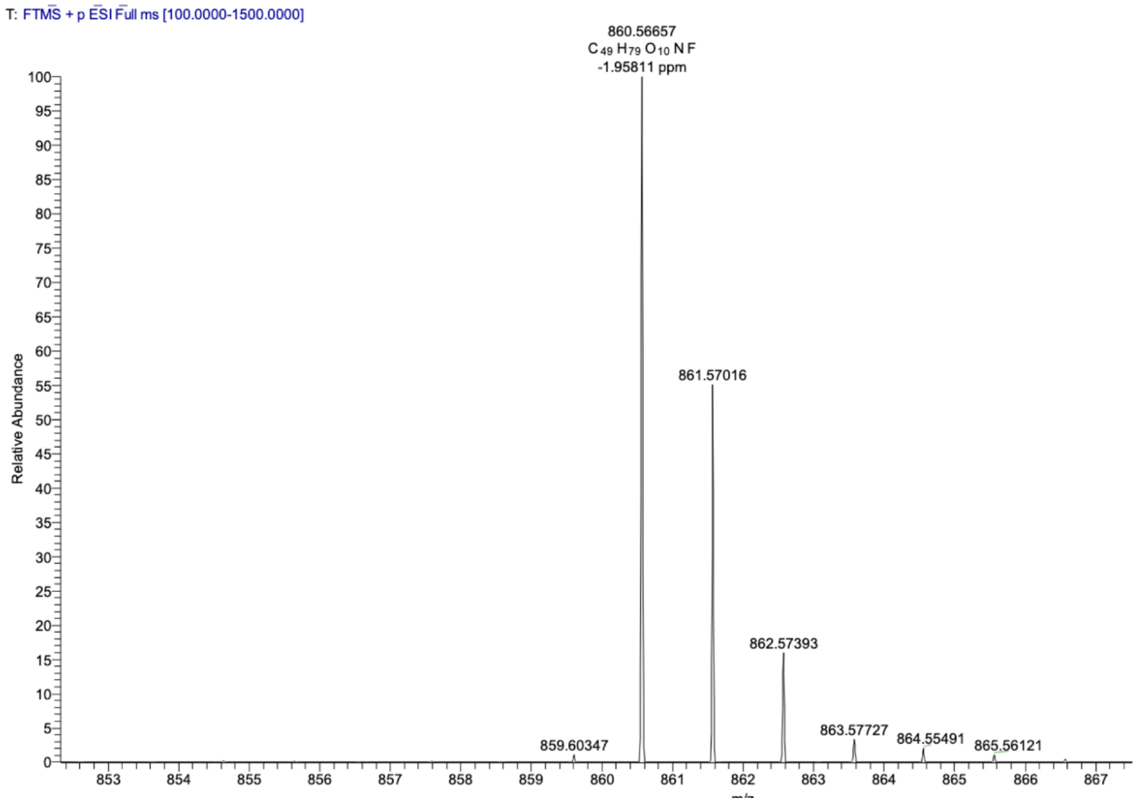

Figure S19. High resolution mass spectrum of compound **5.**

Computational Methods

Monte Carlo conformational searches (MCCS) with an OPLS4 force field were performed using the Schrödinger MacroModel program to locate low energy conformers.[7] The reported energy of each pseudo-transition state was derived from the lowest energy MCCS conformer. For the cation bound structures, initial low energy conformers were located using MCCS and subsequently optimized using the Gaussian 16, Revision C. 01[8] software package. The keyword (integral=grid=ultrafine) was used for all Gaussian 16 calculations. Optimizations were conducted using the Head-Gordon's range-separated hybrid ωB97X-D functional[9] with a 6-31G(d) basis sets and a universal solvent model[10] (SMD) in acetonitrile ($\varepsilon = 37.5$).[11] This functional was selected as it accounts for dispersion and has been shown to provide accurate thermochemical and kinetic energies. Images of the electrostatic potential (kcal/mol) mapped onto electron density (electrons/bohr^3) (isovalue = 0.001, min=-0.3 or -0.35, max=0.3) were calculated at the B3LYP-D3-BJ/MIDIXL level of theory using the program Jaguar of the Schrödinger software package.[12] CYLview[13] was utilized to generate 3D images. The protocol for generation of CYLview 3D images of the pseudo-transition states (not optimized in Gaussian) involved performing a single point energy calculation on the respective lowest energy conformer from MCCS. The Gaussian 16, Revision C. 01 software package[8] was used for these single point calculations performed at the ωB97X-D/6-31G(d) level of theory and the generated .log files rendered in CYLview. GaussView 6.1[14] was used for structure visualization.

Cartesian Coordinates of Computational Structures

Table S1. Energies of all pseudo-transition states derived from lowest energy conformers of MCCS. Pseudo-transition states were constructed by tethering SRD to the desired reaction site via a fluorine linkage to simulate fluorination. This was done for computational efficiency in light of the large size and flexibility of these systems rendering transition state optimizations at DFT impractical.

Structure	Stereochemistry	Energy (kJ/mol)	Relative Energy (kcal/mol)
**3**	*R*	111.566	20.5
**3**	*S*	95.357	16.6
**7**	*R*	25.792	0
**7**	*S*	38.214	3.0

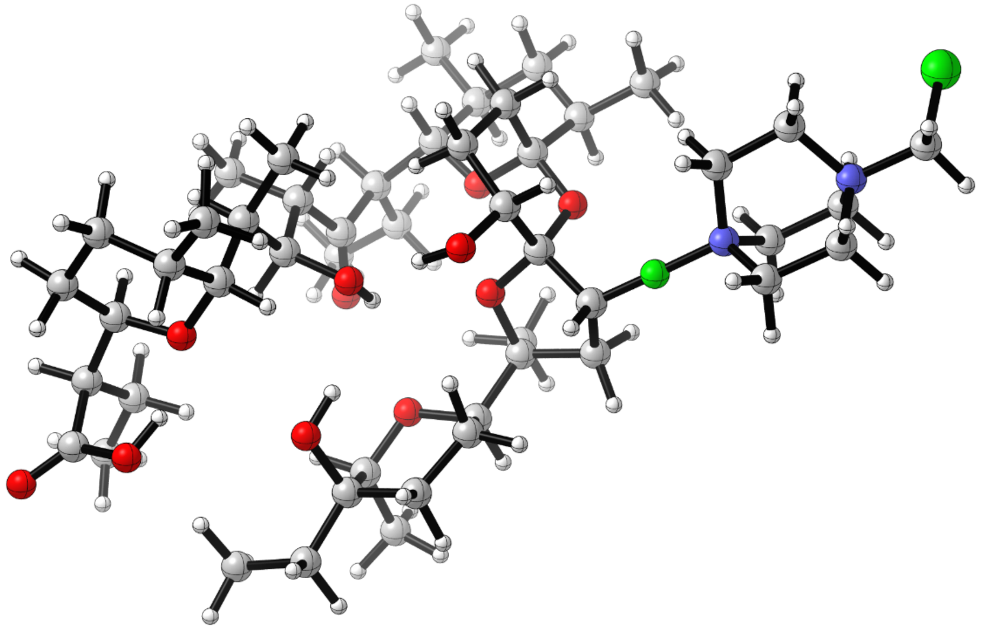

Figure S20. Lowest energy conformer of *R*-**TS-**3

Coordinates of the lowest energy conformer from MCCS listed below.

C	2.88920000	9.43230000	-8.46110000
C	2.00060000	10.32220000	-7.57050000
C	0.83740000	9.55130000	-6.89820000
C	-0.21370000	8.97760000	-7.88930000
C	-1.14730000	7.92800000	-7.24890000
C	-0.32370000	6.93730000	-6.40780000
C	0.47280000	7.71230000	-5.31660000
C	-0.27530000	8.56080000	-4.23090000
C	0.74150000	9.34390000	-3.33610000
C	0.12160000	10.44750000	-2.40260000
C	1.17270000	11.12000000	-1.48820000
C	0.69930000	11.89670000	-0.22970000
C	0.05650000	10.93910000	0.83830000
C	-0.25600000	11.61810000	2.20810000
C	-0.73620000	10.57740000	3.23320000
C	0.26300000	9.40460000	3.32780000
C	0.47210000	8.78450000	1.92470000
C	0.73980000	6.46500000	0.15430000
C	1.93720000	7.15890000	0.86410000
C	3.02640000	6.14100000	1.19650000
C	4.21910000	7.06380000	1.37240000
C	3.99690000	8.16980000	0.32340000
C	4.84560000	8.01650000	-0.98480000

C	4.67060000	6.66920000	-1.72780000
C	5.39960000	6.62510000	-3.09360000
C	5.25270000	7.89610000	-3.96880000
C	5.36700000	9.17410000	-3.06530000
C	2.82720000	11.12420000	-6.52030000
C	3.90610000	12.05260000	-7.10830000
C	-1.12170000	5.70740000	-5.92740000
C	-1.30390000	7.75200000	-3.40340000
C	-0.62650000	11.53560000	-3.21790000
C	1.80600000	12.85940000	0.32310000
C	2.24690000	13.97070000	-0.65140000
C	-1.27850000	12.77090000	2.10310000
C	-0.14070000	8.41300000	4.44030000
C	4.15000000	9.56910000	0.95000000
C	6.22620000	7.87060000	-5.17780000
C	6.15980000	9.07790000	-6.13440000
C	6.79760000	9.59110000	-2.65360000
O	1.38890000	8.51530000	-6.07100000
O	1.49940000	8.40160000	-2.58400000
O	2.36700000	11.04240000	-1.77390000
O	0.92620000	9.81720000	1.06500000
O	1.49960000	7.81920000	2.05090000
O	2.61250000	8.02800000	-0.00320000
O	4.50970000	9.04180000	-1.92520000
O	3.92160000	7.90150000	-4.45160000
O	1.08170000	5.97300000	-1.12810000
H	1.54920000	11.05230000	-8.24380000
H	0.32790000	10.28260000	-6.26950000
H	-0.80200000	9.78990000	-8.31760000
H	0.29470000	8.51180000	-8.73450000
H	-1.66640000	7.38960000	-8.04440000
H	-1.94300000	8.37950000	-6.65460000
H	0.44080000	6.52390000	-7.07150000
H	1.08450000	6.98040000	-4.78410000
H	-0.86470000	9.29680000	-4.76810000
H	1.45780000	9.84650000	-3.99360000
H	-0.59700000	9.97240000	-1.74310000
H	-0.09890000	12.54870000	-0.57810000
H	-0.88100000	10.58240000	0.41220000
H	0.68530000	11.98780000	2.61490000
H	-1.72960000	10.21330000	2.96670000
H	1.23650000	9.81370000	3.60700000
H	0.44710000	5.60520000	0.75940000
H	3.15420000	5.43690000	0.37260000
H	4.19980000	7.47500000	2.38300000
H	5.16610000	6.53940000	1.24900000

H	5.88120000	8.11990000	-0.66050000
H	5.02360000	5.84660000	-1.10690000
H	3.60870000	6.48350000	-1.88600000
H	5.11780000	5.73850000	-3.66490000
H	6.46310000	6.47730000	-2.90060000
H	4.96030000	10.01480000	-3.63300000
H	3.29210000	10.43890000	-5.80730000
H	2.15140000	11.74020000	-5.92420000
H	4.39750000	12.62750000	-6.32270000
H	3.47580000	12.76180000	-7.81640000
H	4.68260000	11.49430000	-7.63290000
H	-0.55940000	5.12180000	-5.19890000
H	-2.08270000	5.96650000	-5.48970000
H	-1.34190000	5.04630000	-6.76690000
H	-0.86530000	6.84110000	-2.99460000
H	-1.70860000	8.32850000	-2.57420000
H	-2.16780000	7.47570000	-4.00290000
H	-1.03050000	12.32730000	-2.58570000
H	0.02720000	12.00920000	-3.95320000
H	-1.48260000	11.12630000	-3.75320000
H	2.67710000	12.28470000	0.64270000
H	1.45980000	13.37860000	1.21440000
H	2.95400000	14.64840000	-0.17140000
H	1.39810000	14.56830000	-0.98540000
H	-1.45740000	13.23140000	3.07600000
H	-0.93320000	13.56360000	1.43890000
H	-2.24050000	12.42260000	1.72430000
H	0.57560000	7.59820000	4.52020000
H	-1.12630000	7.98150000	4.26540000
H	-0.17320000	8.90990000	5.41070000
H	3.47910000	9.70620000	1.79760000
H	5.16970000	9.74410000	1.29020000
H	3.90790000	10.34340000	0.22420000
H	7.25030000	7.77020000	-4.81740000
H	6.03460000	6.96720000	-5.75940000
H	6.85170000	8.95340000	-6.96780000
H	6.40780000	10.01630000	-5.64050000
H	5.16470000	9.18350000	-6.55890000
H	7.34950000	8.77490000	-2.19010000
H	6.76710000	10.42300000	-1.94970000
H	7.38030000	9.91920000	-3.51340000
H	2.14160000	8.90800000	-2.05100000
H	3.33550000	7.97090000	-3.68660000
H	1.22940000	6.74930000	-1.69830000
H	2.74350000	13.57450000	-1.53750000
C	-0.44940000	7.41090000	0.02110000

H	-0.21100000	8.18320000	-0.70160000
H	-1.31410000	6.87340000	-0.36930000
H	-0.87270000	11.05380000	4.20630000
O	3.31160000	9.79030000	-9.55440000
C	-0.77420000	8.06810000	1.35930000
H	-1.61490000	8.75150000	1.26730000
H	-1.08090000	7.30260000	2.07110000
F	2.75580000	5.44770000	2.32400000
H	2.66280000	8.23360000	-7.04130000
O	3.15920000	8.23870000	-7.87740000
C	2.92910000	5.40670000	4.78530000
C	0.96180000	4.49220000	3.72030000
C	3.06360000	3.33380000	3.57260000
C	2.60560000	4.66480000	6.12160000
H	2.50680000	6.41440000	4.79090000
H	4.00600000	5.56510000	4.68670000
C	0.56440000	3.71760000	5.01730000
H	0.58540000	3.97290000	2.83500000
H	0.47410000	5.46940000	3.68900000
H	2.73430000	2.79180000	2.68180000
H	4.14350000	3.44490000	3.44420000
C	2.74690000	2.51060000	4.86320000
H	1.96730000	5.26710000	6.76990000
H	3.50770000	4.39540000	6.67400000
H	0.03930000	2.78620000	4.79790000
H	-0.06270000	4.32570000	5.67110000
H	2.20890000	1.58820000	4.63500000
H	3.65330000	2.25770000	5.41690000
C	1.53280000	2.53520000	7.08570000
H	2.47990000	2.29710000	7.57250000
H	1.02110000	1.62020000	6.78290000
Cl	0.49480000	3.43630000	8.23090000
N	1.84670000	3.35070000	5.80650000
N	2.44070000	4.69890000	3.55380000

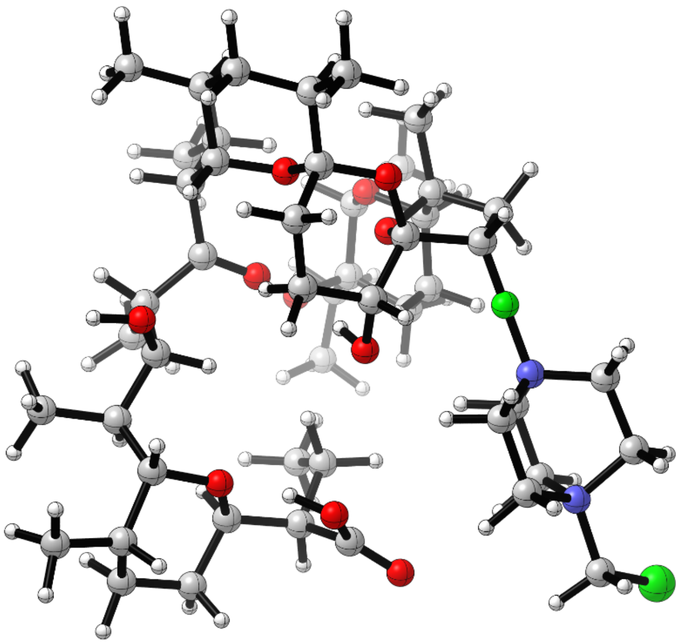

Figure S21. Lowest energy conformer of *S*-**TS-**3

Coordinates of the lowest energy conformer from MCCS listed below.

C	6.79160000	-7.26460000	14.77920000
C	5.34220000	-6.97430000	14.34240000
C	4.23790000	-7.30800000	15.39040000
C	3.84290000	-8.80450000	15.39650000
C	2.89040000	-9.16070000	16.55690000
C	3.44560000	-8.60290000	17.87590000
C	3.63540000	-7.06210000	17.76350000
C	2.41770000	-6.08570000	17.57510000
C	2.93990000	-4.61550000	17.62430000
C	2.04320000	-3.47660000	17.04980000
C	2.90170000	-2.22800000	16.74300000
C	2.64690000	-0.93050000	17.54590000
C	3.74180000	-0.73850000	18.64600000
C	3.56670000	0.50900000	19.55720000
C	4.77190000	0.60300000	20.51680000
C	6.11180000	0.60130000	19.74160000
C	6.17030000	-0.61560000	18.78770000
C	7.25830000	-2.82140000	17.24330000
C	7.40640000	-1.31330000	16.82600000
C	8.74950000	-1.03560000	16.11420000
C	8.36160000	-0.06060000	14.99160000
C	6.84210000	0.16200000	15.07960000
C	6.06850000	0.06020000	13.71710000
C	6.19870000	-1.31080000	12.99170000

C	5.22600000	-1.49420000	11.80380000
C	3.78130000	-1.01540000	12.08030000
C	3.84080000	0.40000000	12.74480000
C	5.21150000	-5.52160000	13.79560000
C	4.07110000	-5.34900000	12.77680000
C	2.70590000	-9.10920000	19.13140000
C	1.21660000	-6.32460000	18.52370000
C	1.20960000	-3.86860000	15.80300000
C	2.44200000	0.30000000	16.61220000
C	1.25750000	0.17940000	15.63430000
C	2.24440000	0.50710000	20.35230000
C	7.32220000	0.74030000	20.68940000
C	6.49520000	1.46500000	15.83770000
C	2.86750000	-1.05740000	10.82610000
C	2.64160000	-2.45030000	10.20140000
C	4.22710000	1.57800000	11.82420000
O	4.61160000	-6.88900000	16.71800000
O	3.21780000	-4.29660000	18.97680000
O	3.75170000	-2.27580000	15.84790000
O	5.00390000	-0.59080000	17.98720000
O	7.29800000	-0.45420000	17.95550000
O	6.46870000	-0.97850000	15.84110000
O	4.67560000	0.34940000	13.90520000
O	3.21580000	-1.86460000	13.06220000
O	6.78940000	-3.59410000	16.15190000
H	5.19170000	-7.63520000	13.48720000
H	3.35720000	-6.75450000	15.07200000
H	3.37350000	-9.06670000	14.44730000
H	4.74360000	-9.41700000	15.45900000
H	2.80680000	-10.24740000	16.62270000
H	1.87070000	-8.81040000	16.38560000
H	4.45880000	-9.00210000	17.97100000
H	4.13430000	-6.75030000	18.68420000
H	2.01630000	-6.26020000	16.58230000
H	3.88900000	-4.59780000	17.08010000
H	1.32830000	-3.19170000	17.82050000
H	1.70420000	-1.08470000	18.06640000
H	3.73200000	-1.63150000	19.27200000
H	3.61150000	1.39540000	18.92380000
H	4.74670000	-0.22320000	21.22970000
H	6.11590000	1.47550000	19.08740000
H	8.23980000	-3.18290000	17.54960000
H	9.47080000	-0.62330000	16.82100000
H	8.90520000	0.87890000	15.09400000
H	8.64990000	-0.47180000	14.02640000
H	6.50090000	0.83640000	13.08470000

H	7.21700000	-1.45280000	12.63810000
H	6.01960000	-2.11140000	13.71010000
H	5.22660000	-2.52810000	11.45970000
H	5.60580000	-0.94000000	10.94460000
H	2.83970000	0.62900000	13.11670000
H	6.13170000	-5.20360000	13.30020000
H	5.06890000	-4.81960000	14.61940000
H	4.04100000	-4.32850000	12.39750000
H	3.09150000	-5.54910000	13.20940000
H	4.19690000	-6.00950000	11.91830000
H	3.05540000	-8.60790000	20.03490000
H	1.62710000	-8.98430000	19.06950000
H	2.87980000	-10.17700000	19.27240000
H	1.52710000	-6.42840000	19.56410000
H	0.49640000	-5.50710000	18.47160000
H	0.66670000	-7.22240000	18.24330000
H	0.63040000	-3.02410000	15.42460000
H	1.84510000	-4.21270000	14.98440000
H	0.49030000	-4.65670000	16.02520000
H	3.35750000	0.48520000	16.04840000
H	2.28160000	1.19990000	17.20450000
H	1.13220000	1.10040000	15.06400000
H	0.32190000	-0.00990000	16.16140000
H	2.17070000	1.38410000	20.99720000
H	1.37760000	0.52890000	19.69130000
H	2.15690000	-0.37550000	20.98740000
H	7.27290000	1.67120000	21.25540000
H	8.26090000	0.74820000	20.13420000
H	7.36920000	-0.07660000	21.40990000
H	7.14270000	1.63600000	16.69570000
H	6.57700000	2.33430000	15.18650000
H	5.47270000	1.42960000	16.21060000
H	1.89380000	-0.63960000	11.08710000
H	3.27350000	-0.39980000	10.05720000
H	1.95740000	-2.38990000	9.35450000
H	2.20550000	-3.14210000	10.92330000
H	3.57120000	-2.88790000	9.83880000
H	3.47440000	1.74170000	11.05330000
H	5.17950000	1.41730000	11.32150000
H	4.30450000	2.50150000	12.39830000
H	2.44330000	-4.52280000	19.49980000
H	3.67080000	-1.68250000	13.90110000
H	5.97060000	-3.15140000	15.88570000
H	1.40570000	-0.62490000	14.91280000
C	6.28980000	-3.06870000	18.40640000
H	5.27510000	-3.09300000	18.02530000

H	6.46080000	-4.05330000	18.84190000
H	4.68550000	1.49910000	21.13440000
O	7.67500000	-7.58140000	13.98660000
C	6.35810000	-1.97700000	19.46910000
H	5.61330000	-2.12850000	20.24800000
H	7.33120000	-1.98700000	19.96070000
F	9.30030000	-2.13150000	15.52520000
H	6.11790000	-6.86780000	16.46610000
O	7.00390000	-7.04840000	16.09760000
C	11.30060000	-3.06650000	14.39690000
C	9.94390000	-4.52210000	15.74350000
C	9.10580000	-3.71340000	13.64110000
C	11.95890000	-4.28570000	13.66680000
H	11.89820000	-2.77370000	15.26380000
H	11.30220000	-2.18740000	13.74780000
C	10.55500000	-5.78840000	15.07090000
H	8.93280000	-4.76350000	16.07590000
H	10.48510000	-4.26780000	16.65740000
H	8.07650000	-3.90680000	13.95320000
H	9.03970000	-2.85320000	12.97310000
C	9.67870000	-4.94730000	12.87870000
H	12.84550000	-4.65180000	14.18720000
H	12.23370000	-4.04080000	12.64000000
H	9.83770000	-6.61070000	15.03980000
H	11.45310000	-6.13280000	15.58570000
H	8.96940000	-5.77380000	12.84210000
H	9.96500000	-4.69480000	11.85740000
C	11.52720000	-6.72490000	12.93790000
H	10.74610000	-7.48870000	12.95190000
H	12.38820000	-7.05060000	13.52280000
Cl	12.03580000	-6.43000000	11.24850000
N	10.94480000	-5.45710000	13.60980000
N	9.89180000	-3.31100000	14.85610000

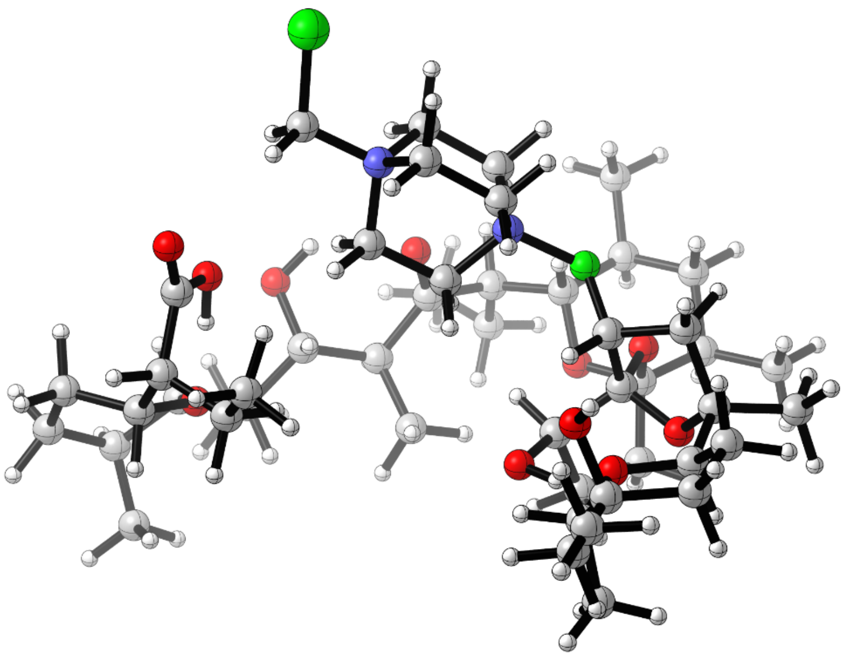

Figure S22. Lowest energy conformer of *R*-**TS-**7

Coordinates of the lowest energy conformer from MCCS listed below.

C	-37.76880000	17.07560000	-13.16850000
C	-38.25920000	17.41140000	-14.58470000
C	-37.54120000	18.63100000	-15.21420000
C	-37.87140000	20.01070000	-14.60900000
C	-37.11030000	21.15800000	-15.30480000
C	-35.73160000	20.76100000	-15.89310000
C	-35.19730000	19.45430000	-15.22950000
C	-33.78160000	18.94700000	-15.63590000
C	-33.31600000	17.74830000	-14.74710000
C	-32.03070000	16.99930000	-15.23310000
C	-31.51760000	15.92190000	-14.23580000
C	-30.05690000	15.41580000	-14.28870000
C	-29.97140000	13.88870000	-14.62280000
C	-28.59910000	13.26760000	-14.21750000
C	-28.52470000	11.82770000	-14.73660000
C	-28.78910000	11.79020000	-16.25390000
C	-30.17980000	12.41160000	-16.54580000
C	-32.87760000	12.75140000	-17.09460000
C	-32.42860000	11.41300000	-16.46420000
C	-33.39580000	10.85870000	-15.40490000
C	-33.15110000	9.37370000	-15.54910000
C	-32.99010000	9.17260000	-17.05850000
C	-34.31520000	9.03060000	-17.88790000

C	-35.11880000	7.73560000	-17.58680000
C	-36.52980000	7.70850000	-18.21110000
C	-37.31850000	9.02460000	-18.03390000
C	-36.39680000	10.22020000	-18.46300000
C	-38.20410000	16.17410000	-15.53130000
C	-39.10570000	14.99480000	-15.11980000
C	-35.84480000	20.58200000	-17.42690000
C	-32.72160000	20.08000000	-15.71370000
C	-32.24220000	16.39000000	-16.64190000
C	-29.06420000	16.32760000	-15.08090000
C	-28.69240000	17.62870000	-14.34330000
C	-28.33380000	13.28590000	-12.69550000
C	-28.59610000	10.35320000	-16.78510000
C	-31.95360000	8.08570000	-17.39600000
C	-38.68520000	9.01890000	-18.77000000
C	-39.71320000	7.96270000	-18.30700000
C	-36.14150000	10.37100000	-19.97800000
O	-36.13300000	18.36560000	-15.28330000
O	-33.12920000	18.22980000	-13.42800000
O	-32.25920000	15.50090000	-13.34500000
O	-30.20160000	13.73240000	-16.03250000
O	-31.14800000	11.59680000	-15.88550000
O	-32.37890000	10.40050000	-17.44640000
O	-35.14300000	10.20000000	-17.76920000
O	-37.59590000	9.20040000	-16.65450000
O	-34.24120000	12.76940000	-17.48160000
H	-39.30880000	17.68500000	-14.46980000
H	-37.89000000	18.71370000	-16.24600000
H	-38.94360000	20.19910000	-14.68160000
H	-37.65420000	20.00020000	-13.53980000
H	-36.92860000	21.90940000	-14.53300000
H	-37.71550000	21.71680000	-16.02190000
H	-35.03490000	21.57330000	-15.67960000
H	-35.13900000	19.70470000	-14.16790000
H	-33.90940000	18.56280000	-16.64590000
H	-34.12410000	17.01370000	-14.70390000
H	-31.24950000	17.74920000	-15.30110000
H	-29.75810000	15.49300000	-13.24550000
H	-30.75870000	13.34950000	-14.09050000
H	-27.80130000	13.80400000	-14.72790000
H	-29.24720000	11.19930000	-14.21050000
H	-28.05600000	12.42820000	-16.75120000
H	-32.75810000	13.51360000	-16.32550000
H	-34.42950000	11.10610000	-15.64110000
H	-32.23740000	9.09290000	-15.02260000
H	-33.95050000	8.77750000	-15.11410000

H	-33.99290000	8.98130000	-18.92940000
H	-34.55980000	6.86420000	-17.92970000
H	-35.22060000	7.61240000	-16.50950000
H	-37.10160000	6.85620000	-17.84340000
H	-36.43830000	7.50650000	-19.27930000
H	-36.88690000	11.14580000	-18.15320000
H	-37.17370000	15.82670000	-15.64050000
H	-38.50690000	16.47760000	-16.53600000
H	-40.14830000	15.30590000	-15.03710000
H	-38.81520000	14.56430000	-14.16200000
H	-39.06360000	14.19530000	-15.86080000
H	-36.50700000	19.76270000	-17.70450000
H	-36.22810000	21.48610000	-17.90220000
H	-34.87560000	20.38120000	-17.88300000
H	-31.75040000	19.71050000	-16.03970000
H	-32.99980000	20.85750000	-16.42430000
H	-32.58050000	20.55670000	-14.74250000
H	-31.39640000	15.78280000	-16.96240000
H	-32.34920000	17.16480000	-17.40090000
H	-33.13540000	15.76530000	-16.68600000
H	-29.44390000	16.55520000	-16.07710000
H	-28.13130000	15.80530000	-15.28180000
H	-28.17070000	17.41300000	-13.40980000
H	-29.55820000	18.24180000	-14.09440000
H	-28.23480000	14.30020000	-12.30930000
H	-29.13370000	12.79320000	-12.14110000
H	-27.40250000	12.77240000	-12.45090000
H	-27.60050000	9.97590000	-16.54830000
H	-28.70500000	10.29150000	-17.86710000
H	-29.31690000	9.67090000	-16.33660000
H	-32.29510000	7.09770000	-17.08900000
H	-31.74700000	8.05580000	-18.46590000
H	-31.00520000	8.27210000	-16.89520000
H	-39.14220000	10.00570000	-18.67510000
H	-38.51840000	8.87770000	-19.83850000
H	-40.63430000	8.04560000	-18.88580000
H	-39.98530000	8.08830000	-17.25870000
H	-39.34420000	6.94580000	-18.44250000
H	-35.46920000	11.20690000	-20.17250000
H	-37.06470000	10.57220000	-20.52060000
H	-35.69300000	9.47890000	-20.41250000
H	-32.82320000	17.49030000	-12.88980000
H	-38.15240000	8.46680000	-16.38340000
H	-34.44450000	11.89290000	-17.85010000
H	-28.02450000	18.24170000	-14.95010000
C	-31.94760000	13.06910000	-18.28230000

H	-32.33110000	12.59790000	-19.18850000
H	-31.96190000	14.13860000	-18.48510000
H	-27.55150000	11.39450000	-14.49710000
O	-38.47030000	16.54410000	-12.31720000
C	-30.49820000	12.56710000	-18.04830000
H	-30.37350000	11.59570000	-18.52440000
H	-29.76570000	13.23600000	-18.50070000
F	-33.11470000	11.27210000	-14.11970000
H	-36.18140000	17.75380000	-13.85250000
O	-36.44670000	17.32140000	-13.01720000
C	-34.63650000	13.34360000	-13.89990000
C	-35.24410000	11.41470000	-12.63260000
C	-33.26710000	12.64180000	-12.04330000
C	-35.45680000	14.29000000	-12.97490000
H	-35.25250000	13.02280000	-14.74360000
H	-33.81180000	13.88950000	-14.35770000
C	-36.11930000	12.27540000	-11.66060000
H	-34.87430000	10.52160000	-12.12270000
H	-35.85470000	11.03290000	-13.45540000
H	-32.85320000	11.77920000	-11.51580000
H	-32.39450000	13.16920000	-12.43260000
C	-34.05210000	13.56010000	-11.05560000
H	-36.48390000	14.41570000	-13.31860000
H	-34.98440000	15.26970000	-12.89190000
H	-36.14660000	11.85300000	-10.65540000
H	-37.14340000	12.39240000	-12.01970000
H	-34.07670000	13.14290000	-10.04880000
H	-33.63240000	14.56710000	-11.01050000
N	-34.07970000	12.14900000	-13.20160000
N	-35.51430000	13.69880000	-11.54610000
C	-36.35830000	14.62070000	-10.63260000
H	-35.92030000	15.62030000	-10.66400000
H	-37.37140000	14.64730000	-11.03490000
Cl	-36.41680000	14.04220000	-8.94130000

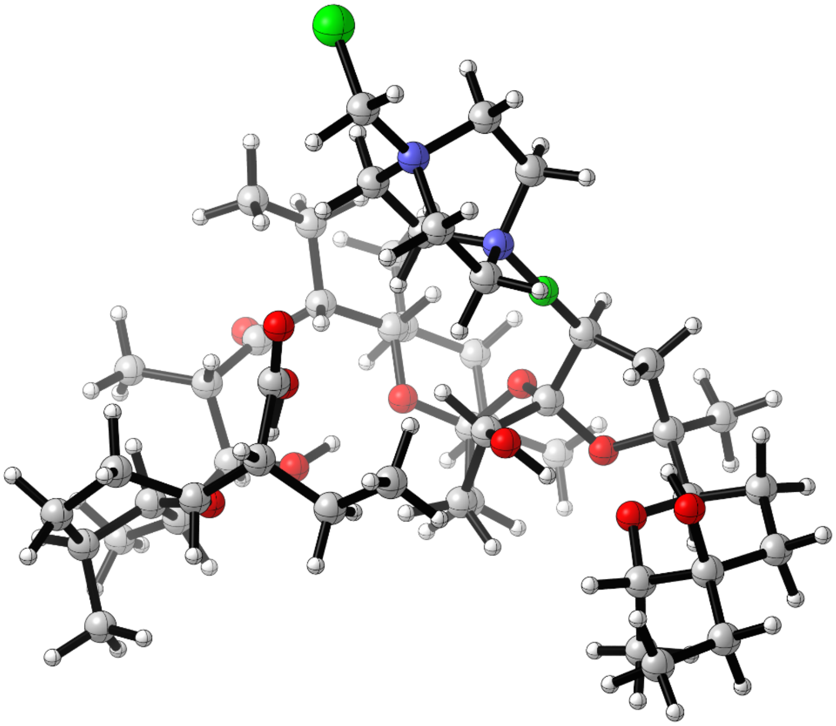

Figure S23. Lowest energy conformer of *S*-**TS-**7

Coordinates of the lowest energy conformer from MCCS listed below.

C	-35.97600000	16.17650000	-14.07400000
C	-36.90210000	16.58050000	-15.22770000
C	-36.47710000	17.90600000	-15.89740000
C	-36.76050000	19.20030000	-15.10400000
C	-36.32170000	20.46850000	-15.86880000
C	-35.12600000	20.25820000	-16.83360000
C	-34.31540000	18.98760000	-16.43050000
C	-32.97060000	18.70960000	-17.17540000
C	-32.04010000	17.66500000	-16.47130000
C	-31.65860000	17.88910000	-14.97200000
C	-30.54160000	16.91650000	-14.53730000
C	-30.95580000	15.56470000	-13.92230000
C	-30.43910000	14.31350000	-14.70170000
C	-28.92640000	14.23890000	-15.06780000
C	-28.70240000	12.94930000	-15.87030000
C	-29.61330000	12.90880000	-17.11250000
C	-31.09590000	13.10040000	-16.69170000
C	-33.79040000	12.63620000	-16.11480000
C	-32.77340000	11.46920000	-15.95140000
C	-32.97190000	10.58230000	-14.71230000
C	-33.86210000	9.47600000	-15.24770000

C	-33.43190000	9.30270000	-16.70650000
C	-34.61130000	9.07640000	-17.71100000
C	-35.33770000	7.72090000	-17.50640000
C	-36.65530000	7.59420000	-18.30990000
C	-37.55160000	8.85440000	-18.27500000
C	-36.66590000	10.11460000	-18.55260000
C	-37.08820000	15.43410000	-16.26460000
C	-37.85310000	14.21060000	-15.73130000
C	-35.64240000	20.13510000	-18.28950000
C	-32.19050000	19.97690000	-17.62270000
C	-31.27460000	19.34620000	-14.59510000
C	-30.70650000	15.52500000	-12.38730000
C	-31.63560000	16.45370000	-11.58440000
C	-27.97030000	14.26230000	-13.85650000
C	-29.34260000	11.62230000	-17.92710000
C	-32.27230000	8.30210000	-16.87170000
C	-38.79410000	8.71030000	-19.19060000
C	-39.77020000	9.90560000	-19.19550000
C	-36.18290000	10.30620000	-20.00520000
O	-35.13040000	17.80740000	-16.37130000
O	-32.68030000	16.40640000	-16.57410000
O	-29.35740000	17.23710000	-14.62270000
O	-31.20370000	14.28180000	-15.92140000
O	-31.45810000	11.97880000	-15.89790000
O	-32.88420000	10.57530000	-17.03150000
O	-35.57880000	10.13680000	-17.61760000
O	-38.00350000	8.99930000	-16.94190000
O	-35.14660000	12.23700000	-16.02350000
H	-37.87930000	16.76180000	-14.77980000
H	-37.10000000	18.02090000	-16.78620000
H	-37.82660000	19.27080000	-14.88460000
H	-36.26830000	19.14190000	-14.13250000
H	-36.00760000	21.18950000	-15.11110000
H	-37.14620000	20.99490000	-16.35460000
H	-34.47440000	21.13040000	-16.76240000
H	-34.04330000	19.16660000	-15.39060000
H	-33.26920000	18.24420000	-18.11710000
H	-31.12380000	17.59180000	-17.06530000
H	-32.53010000	17.63880000	-14.36930000
H	-32.03900000	15.52040000	-14.03330000
H	-30.70180000	13.43890000	-14.10250000
H	-28.68760000	15.07340000	-15.72920000
H	-28.87950000	12.07630000	-15.23800000
H	-29.36060000	13.76320000	-17.74350000
H	-33.61050000	13.34500000	-15.30660000
H	-32.00440000	10.19790000	-14.38460000

H	-33.77230000	8.55580000	-14.67110000
H	-34.90700000	9.77920000	-15.18590000
H	-34.15730000	9.09570000	-18.70310000
H	-34.67310000	6.89850000	-17.77390000
H	-35.55840000	7.58430000	-16.44740000
H	-37.22750000	6.72020000	-17.99290000
H	-36.41600000	7.37540000	-19.35160000
H	-37.26430000	10.99760000	-18.31650000
H	-36.12060000	15.11750000	-16.66160000
H	-37.63820000	15.80850000	-17.13050000
H	-38.85800000	14.47970000	-15.40430000
H	-37.34060000	13.74710000	-14.89060000
H	-37.95220000	13.44850000	-16.50560000
H	-34.83130000	20.00220000	-19.00420000
H	-36.31810000	19.29200000	-18.42570000
H	-36.18120000	21.03370000	-18.59260000
H	-31.22540000	19.72000000	-18.06120000
H	-32.72960000	20.53410000	-18.38800000
H	-32.00430000	20.67490000	-16.80940000
H	-30.94620000	19.41730000	-13.55640000
H	-32.11190000	20.03530000	-14.68580000
H	-30.45560000	19.72030000	-15.21230000
H	-29.67470000	15.79330000	-12.15660000
H	-30.81980000	14.50660000	-12.01690000
H	-31.43750000	17.50340000	-11.80560000
H	-32.68540000	16.26400000	-11.80860000
H	-28.25060000	13.52960000	-13.09900000
H	-26.94360000	14.04890000	-14.15760000
H	-27.94380000	15.24570000	-13.38930000
H	-28.28140000	11.51390000	-18.15510000
H	-29.86740000	11.62120000	-18.88160000
H	-29.65320000	10.73090000	-17.38010000
H	-32.56470000	7.29250000	-16.58500000
H	-31.92440000	8.27310000	-17.90450000
H	-31.41650000	8.58630000	-16.25900000
H	-38.47230000	8.51640000	-20.21430000
H	-39.34850000	7.81990000	-18.88880000
H	-40.63620000	9.69730000	-19.82480000
H	-39.30300000	10.81320000	-19.57710000
H	-40.14190000	10.11740000	-18.19200000
H	-35.53350000	11.17830000	-20.08330000
H	-37.02350000	10.46770000	-20.67990000
H	-35.63210000	9.44460000	-20.37920000
H	-32.04580000	15.69180000	-16.38780000
H	-37.23790000	9.28550000	-16.43600000
H	-35.28640000	11.56670000	-16.71390000

H	-31.49140000	16.31970000	-10.51180000
C	-33.53840000	13.32750000	-17.46520000
H	-34.12250000	12.84020000	-18.24760000
H	-33.91540000	14.34840000	-17.42610000
H	-27.65310000	12.87200000	-16.16270000
O	-36.38500000	15.88280000	-12.95450000
C	-32.04990000	13.29150000	-17.89020000
H	-31.90030000	12.45800000	-18.57470000
H	-31.77090000	14.19610000	-18.43130000
F	-33.56500000	11.20230000	-13.66480000
H	-34.62320000	16.53480000	-15.34070000
O	-34.67320000	16.14550000	-14.44580000
C	-35.68720000	12.09160000	-12.76490000
C	-34.11930000	11.05670000	-11.25840000
C	-33.63960000	13.21430000	-12.22550000
C	-36.43980000	12.75960000	-11.57360000
H	-36.15260000	11.14190000	-13.03770000
H	-35.78520000	12.71310000	-13.65720000
C	-34.80430000	11.71430000	-10.01520000
H	-33.06130000	10.87190000	-11.05610000
H	-34.54420000	10.06780000	-11.44790000
H	-32.56820000	13.09090000	-12.06130000
H	-33.71900000	13.83570000	-13.12140000
C	-34.30150000	13.94910000	-11.01790000
H	-37.21450000	12.10960000	-11.16400000
H	-36.90290000	13.70280000	-11.86480000
H	-34.08600000	11.92100000	-9.22070000
H	-35.60670000	11.09680000	-9.60780000
H	-33.58430000	14.14110000	-10.21940000
H	-34.75180000	14.89790000	-11.31130000
N	-34.22410000	11.86410000	-12.52130000
N	-35.43360000	13.06630000	-10.43640000
C	-36.17470000	13.76880000	-9.27240000
H	-36.59020000	14.69940000	-9.66480000
H	-36.97400000	13.10780000	-8.93470000
Cl	-35.09140000	14.13900000	-7.89780000

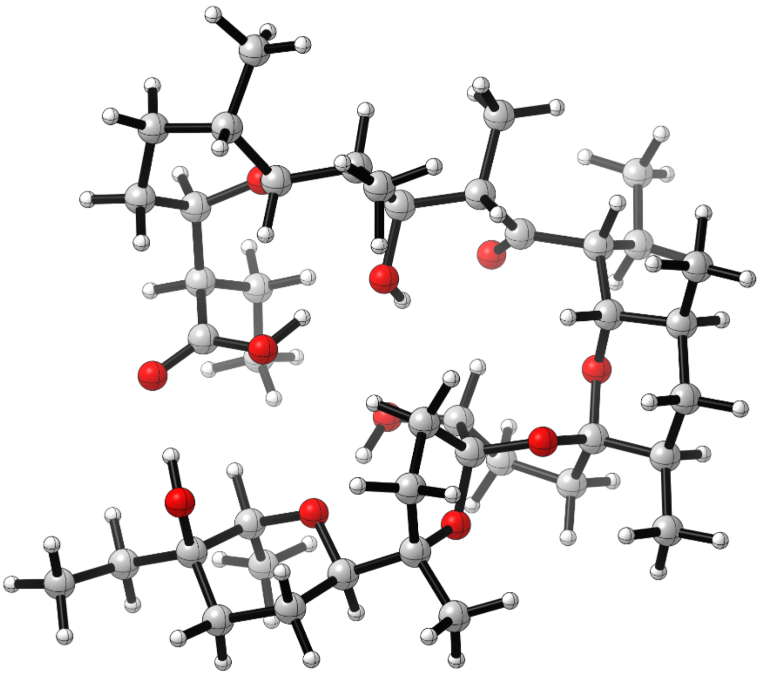

Figure S24. Structure of **7** optimized at the ωB97X-D/6-31G(d) level of theory.

Zero-point correction=	1.134283 (Hartree/Particle)
Thermal correction to Energy=	1.188994
Thermal correction to Enthalpy=	1.189938
Thermal correction to Gibbs Free Energy=	1.049384
Sum of electronic and zero-point Energies=	-2469.588059
Sum of electronic and thermal Energies=	-2469.533348
Sum of electronic and thermal Enthalpies=	-2469.532404
Sum of electronic and thermal Free Energies=	-2469.672958

Number of Imaginary Frequencies = 0

0 1

C	3.30628100	1.43035400	0.50355500
C	3.08288200	2.57374600	1.48364700
C	2.79975500	3.93011800	0.79295300
C	3.77338200	4.26152800	-0.34476100
C	3.18337000	5.39889800	-1.17117400
C	1.85361800	4.98427800	-1.84115000
C	1.20221300	3.85238800	-0.99985200
C	-0.31249500	3.69466800	-1.19742900
C	-0.96230200	2.87504600	-0.07198900
C	-2.47420000	2.67634300	-0.27487000
C	-3.08382000	1.82043000	0.83245000
C	-4.45090500	1.19646100	0.55008200

C	-4.23866200	-0.16239500	-0.15705200
C	-5.47188600	-0.71588300	-0.88692300
C	-5.12742000	-2.11875100	-1.40817900
C	-4.55535300	-3.04181600	-0.32429200
C	-3.37195700	-2.34060900	0.36127100
C	-1.01397500	-1.31718700	1.16937000
C	-1.03011400	-1.99966600	-0.20232900
C	-0.28441100	-1.28091600	-1.32650300
C	0.30915600	-2.42137000	-2.14862200
C	0.50448400	-3.55801500	-1.12656200
C	1.90764700	-3.61088300	-0.49105700
C	3.04046000	-3.97265800	-1.44909100
C	4.40531300	-3.83641700	-0.76789900
C	4.56472100	-2.45623300	-0.12086400
C	3.36325800	-2.19840300	0.81608300
C	2.04056600	2.24422400	2.56230300
C	2.36422300	0.97641700	3.34948500
C	0.98016700	6.22225700	-2.04777300
C	-0.58409700	3.11422400	-2.59000300
C	-3.21862000	4.02405800	-0.31004800
C	-5.29470500	1.09806900	1.82283800
C	-5.69443500	2.47035400	2.36284200
C	-5.93643000	0.18651300	-2.02945900
C	-4.18981200	-4.40811900	-0.90017800
C	0.12129600	-4.92128900	-1.69380600
C	5.88585600	-2.31835700	0.65965700
C	7.12836000	-2.37951500	-0.22608900
C	3.32699300	-2.99917800	2.11801100
O	1.44433800	4.07111800	0.38333600
O	-0.30482400	1.61167100	0.00203400
O	-2.49982100	1.64545200	1.88557800
O	-3.81298800	-1.07204900	0.83626300
O	-2.37558500	-2.17975600	-0.62772900
O	-0.40118800	-3.25569200	-0.05595100
O	2.14255700	-2.33761800	0.09193700
O	4.51991700	-1.52613900	-1.18588800
O	0.27506800	-0.88319700	1.53962300
H	4.05973400	2.68967600	1.96701200
H	2.91984100	4.68978500	1.57640900
H	4.74141200	4.53775000	0.08533100
H	3.95922800	3.38852000	-0.98166500
H	3.89345000	5.74226900	-1.93055700
H	2.99633000	6.25279600	-0.50622900
H	2.08623400	4.55525800	-2.82458200
H	1.67005200	2.90762600	-1.30364300
H	-0.76057100	4.69093000	-1.12804700

H	-0.80672500	3.41011200	0.87076500
H	-2.65162300	2.14797400	-1.22036100
H	-4.95987200	1.84094500	-0.17632000
H	-3.43797600	-0.05061900	-0.90717200
H	-6.28281600	-0.81539700	-0.15271900
H	-4.39404700	-2.03049700	-2.22126400
H	-5.31764700	-3.16990100	0.45617000
H	-1.63556200	-0.42888200	1.08690000
H	0.51090600	-0.66616200	-0.91062800
H	-0.39245200	-2.74999700	-2.92275900
H	1.24379900	-2.13317600	-2.63382600
H	1.85072900	-4.36758800	0.30853600
H	2.90500900	-4.99815800	-1.80973200
H	3.01616700	-3.30280100	-2.31325000
H	5.19847900	-3.97755100	-1.50834200
H	4.53090600	-4.61567200	-0.00517900
H	3.38410600	-1.13842900	1.07942300
H	1.04896800	2.15842700	2.10971300
H	1.98763900	3.10451300	3.24092600
H	3.37129000	1.02153400	3.77986900
H	2.29346100	0.08905800	2.71398000
H	1.64763900	0.83707000	4.16383000
H	0.10816000	6.02454000	-2.67977600
H	0.62835500	6.61061300	-1.08463600
H	1.56309300	7.01368400	-2.53178700
H	-1.64059700	3.19129600	-2.86535300
H	-0.01367700	3.64568800	-3.35791700
H	-0.29629900	2.05815200	-2.62698400
H	-4.30422100	3.89204600	-0.28761400
H	-2.97629800	4.58255100	-1.21819100
H	-2.93933100	4.63617200	0.55452400
H	-4.72248400	0.54696800	2.57278100
H	-6.19545600	0.51132200	1.61156400
H	-6.27197100	3.03764400	1.62230900
H	-4.81248000	3.06198000	2.63120400
H	-5.10680900	0.40838300	-2.71290100
H	-6.72077700	-0.30778500	-2.61244400
H	-6.34562100	1.13697500	-1.67191200
H	-5.05158700	-4.84765900	-1.41431200
H	-3.87391400	-5.10890000	-0.12151700
H	-3.37162900	-4.30801600	-1.61949300
H	0.70232600	-5.15452300	-2.59159500
H	0.28059800	-5.71596300	-0.95715800
H	-0.93926400	-4.90889900	-1.96001500
H	5.86362100	-1.35167800	1.18164000
H	5.94355600	-3.09566900	1.43154800

H	8.02686800	-2.14376900	0.35320000
H	7.04266700	-1.66203800	-1.04600400
H	7.26736600	-3.37547800	-0.66044400
H	2.36475200	-2.82836200	2.61215900
H	4.11727000	-2.66338900	2.79489300
H	3.44863400	-4.07618500	1.96600900
H	-0.56251000	1.17462800	0.82910100
H	4.46424600	-0.63120100	-0.80149100
H	0.91893300	-1.49100200	1.12260600
H	-6.31132000	2.37276800	3.26152200
C	-1.65788300	-2.24120700	2.21075800
H	-0.89024000	-2.92229400	2.58767600
H	-2.00335800	-1.62731400	3.04631700
H	-6.02607400	-2.57793800	-1.83920400
O	4.42097800	0.98730200	0.28374700
C	-2.81977500	-3.04703900	1.59517600
H	-3.63358100	-3.18759700	2.31245000
H	-2.46216300	-4.03071800	1.28395200
H	1.38933300	1.30447000	0.08880300
O	2.26743700	0.90386000	-0.12709000
H	-0.96577800	-0.63933800	-1.88813400

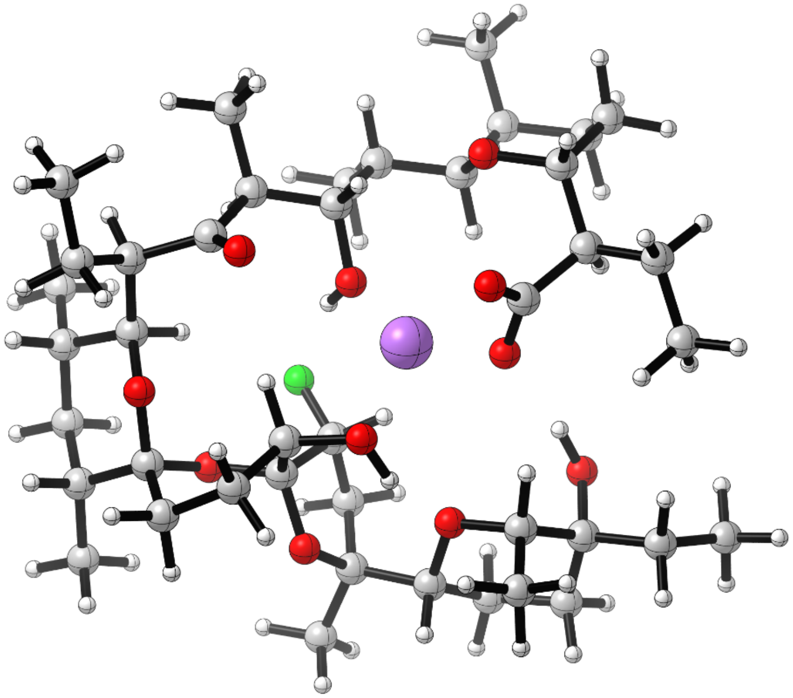

Figure S25. Structure of $[Li]^+$-**4** optimized at the (SMD = MeCN) ωB97X-D/6-31G(d) level of theory.

Zero-point correction=	1.113097 (Hartree/Particle)
Thermal correction to Energy=	1.169365

Thermal correction to Enthalpy=	1.170310
Thermal correction to Gibbs Free Energy=	1.027312
Sum of electronic and zero-point Energies=	-2575.913003
Sum of electronic and thermal Energies=	-2575.856735
Sum of electronic and thermal Enthalpies=	-2575.855791
Sum of electronic and thermal Free Energies=	-2575.998788

Number of Imaginary Frequencies = 0

Charge = 0, Multiplicity = 1

C	2.83932400	1.16974800	1.06840600
C	3.97424100	2.09192800	0.64915000
C	3.53006500	3.57446600	0.65666000
C	4.40506000	4.43687400	-0.25862700
C	4.06033700	4.18760800	-1.72834800
C	2.55704700	4.36784000	-1.99570400
C	1.77086500	3.48042900	-1.00467000
C	0.24447600	3.66675300	-1.07749000
C	-0.49211800	2.88805200	0.03234300
C	-2.00962600	3.11036100	0.03473700
C	-2.71504200	2.28783400	1.10848400
C	-4.15778900	1.89348200	0.78531800
C	-4.15645800	0.58892100	-0.04745700
C	-5.42131900	0.36973000	-0.88940800
C	-5.38491100	-1.04792600	-1.47756800
C	-5.12691300	-2.13252600	-0.42455100
C	-3.85725100	-1.77449000	0.35814200
C	-1.38059000	-1.35093900	1.31945300
C	-1.47388800	-1.94288700	-0.09044300
C	-0.55176600	-1.33190900	-1.16636800
C	-0.28154200	-2.49408500	-2.08926400
C	-0.20814300	-3.68323600	-1.12026400
C	1.19640000	-3.95332400	-0.53103600
C	2.28275200	-4.22802100	-1.56924100
C	3.67197600	-4.28772000	-0.92840700
C	3.94386700	-3.05933200	-0.04740100
C	2.79286800	-2.90664500	0.96403600
C	5.18889500	1.87570200	1.56748700
C	5.64300100	0.41766600	1.60932000
C	2.14570600	5.84213100	-1.92773700
C	-0.27273500	3.32351900	-2.47887100
C	-2.38984000	4.58421800	0.25778900
C	-5.02436200	1.81497700	2.04371800
C	-5.27105600	3.18943600	2.66271900
C	-5.55547000	1.40480900	-2.00554600
C	-5.07986900	-3.52202900	-1.05515700

C	-0.78130800	-4.95749400	-1.72393500
C	5.28794700	-3.17870600	0.69513600
C	6.50797300	-3.09305900	-0.21786100
C	2.72889300	-3.95174100	2.07356000
O	2.15486500	3.77031900	0.33428600
O	-0.22498800	1.48814400	-0.04590200
O	-2.17905400	1.99914800	2.16263500
O	-3.98594700	-0.46973800	0.88613800
O	-2.78399900	-1.83904500	-0.57962200
O	-1.05287100	-3.28951100	-0.01265300
O	1.55038600	-2.81791100	0.25394300
O	3.95453200	-1.92251100	-0.90331400
O	-0.03523600	-1.30151000	1.77271900
H	4.26079000	1.79025700	-0.36334800
H	3.60215100	3.93667100	1.68712800
H	4.23756600	5.49142500	-0.01040900
H	5.46507500	4.23098000	-0.07340600
H	4.35402700	3.16849100	-2.01048200
H	4.63514200	4.86661700	-2.36936000
H	2.35844900	3.99841400	-3.00862600
H	2.00124700	2.42676300	-1.22669000
H	0.04194900	4.72526900	-0.88029000
H	-0.08390500	3.19266900	0.99681300
H	-2.42652200	2.79317900	-0.92927500
H	-4.55186100	2.67725900	0.12814500
H	-3.29590200	0.59284200	-0.73316100
H	-6.29168700	0.44164500	-0.22317700
H	-4.59854600	-1.09899300	-2.24317300
H	-5.94147000	-2.10094200	0.31100900
H	-1.72024900	-0.31771600	1.26866100
H	0.36929100	-0.93713200	-0.73572200
H	-1.14057100	-2.61683000	-2.75677400
H	0.61410500	-2.35744000	-2.69419400
H	1.09329100	-4.82286700	0.13395400
H	2.06648600	-5.16841000	-2.08655700
H	2.28469700	-3.42844000	-2.31455900
H	4.43021400	-4.34088500	-1.71670000
H	3.77374000	-5.19664300	-0.32341800
H	2.88626500	-1.91778200	1.42544500
H	4.93466500	2.21129400	2.58067600
H	6.01568100	2.50708100	1.22147200
H	5.81041300	0.02406900	0.59997000
H	4.88696400	-0.21471300	2.08873600
H	6.57401500	0.30963200	2.17636300
H	2.84438400	6.46166500	-2.50189400
H	1.14579700	6.00409400	-2.34451700

H	2.14049400	6.20806400	-0.89535800
H	-1.34970300	3.49498300	-2.56660000
H	0.20746400	3.94287300	-3.24124200
H	-0.07451200	2.27553400	-2.73621700
H	-3.47023400	4.69970900	0.39481700
H	-2.10054600	5.19545300	-0.60158500
H	-1.88999900	4.97763900	1.14996600
H	-4.53366100	1.15854900	2.76729800
H	-5.98489900	1.35157900	1.79230600
H	-5.78959000	3.85232300	1.95937800
H	-4.32973700	3.67316800	2.94815700
H	-4.65293800	1.42168000	-2.63007100
H	-6.40422800	1.15703500	-2.65299800
H	-5.72051900	2.41653900	-1.62128700
H	-6.00587200	-3.71453000	-1.60859900
H	-4.97339900	-4.31063900	-0.30303500
H	-4.24159300	-3.60176600	-1.75498300
H	-0.22706500	-5.24671600	-2.62231100
H	-0.73875800	-5.78364200	-1.00665000
H	-1.82586400	-4.78964100	-2.00199200
H	5.33883000	-2.37513300	1.44105400
H	5.31219700	-4.12394700	1.25054300
H	7.42990100	-3.08224900	0.37392900
H	6.47540400	-2.18101000	-0.82094900
H	6.56509600	-3.94794100	-0.90128600
H	1.79168600	-3.83239900	2.62818800
H	3.55320900	-3.80461700	2.77689300
H	2.77899100	-4.97976100	1.70254300
H	-0.62864800	1.11921300	-0.84664700
H	3.53454500	-1.16489900	-0.44825400
H	0.46446400	-2.03829000	1.34953100
H	-5.88897500	3.10826000	3.56378100
C	-2.27831900	-2.14496600	2.27565000
H	-1.70482700	-2.99275800	2.66079000
H	-2.53209900	-1.50215100	3.12268000
H	-6.33584600	-1.25482400	-1.98358400
O	2.52820100	0.21048700	0.29288500
C	-3.54917700	-2.65113900	1.56556500
H	-3.40045500	-3.67430800	1.21356300
H	-4.40952200	-2.64583500	2.24010200
F	-1.17951900	-0.27456500	-1.84151900
O	2.22395800	1.38497700	2.14586200
Li	0.76306500	0.39512000	1.23981000

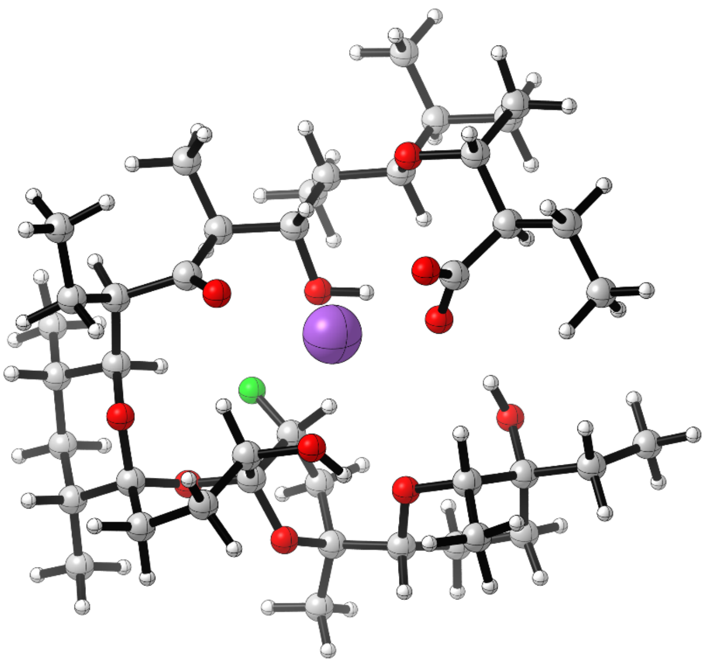

Figure S26. Structure of [Na]$^+$-**4** optimized at the (SMD = MeCN) ωB97X-D/6-31G(d) level of theory.

Zero-point correction=	1.112323 (Hartree/Particle)
Thermal correction to Energy=	1.169058
Thermal correction to Enthalpy=	1.170002
Thermal correction to Gibbs Free Energy=	1.025929
Sum of electronic and zero-point Energies=	-2730.648420
Sum of electronic and thermal Energies=	-2730.591685
Sum of electronic and thermal Enthalpies=	-2730.590741
Sum of electronic and thermal Free Energies=	-2730.734814

Number of Imaginary Frequencies = 0

Charge = 0, Multiplicity = 1

C	-3.19102300	-0.42420200	1.20937600
C	-4.57006400	-0.92375400	0.77351900
C	-4.55271100	-2.46996900	0.71674900
C	-5.68985800	-3.06221400	-0.12175400
C	-5.40348200	-2.93272200	-1.61908000
C	-4.02925600	-3.51793100	-1.98403800
C	-2.97552600	-2.83126400	-1.09514500
C	-1.54045300	-3.36410100	-1.25811600
C	-0.58693800	-2.68618400	-0.25215900
C	0.83884000	-3.24902100	-0.25166100
C	1.70900600	-2.63167000	0.83854500
C	3.22120900	-2.82718300	0.70326200

C	3.84644100	-1.61900600	-0.03753000
C	5.14329900	-1.92738600	-0.79662100
C	5.68514400	-0.60297400	-1.35505500
C	5.82546900	0.49864900	-0.29661600
C	4.49606200	0.65642500	0.45695500
C	2.04622700	1.14420800	1.41072000
C	2.29584600	1.60078000	-0.03357800
C	1.27986000	1.13121400	-1.10303700
C	1.24778400	2.28877900	-2.07562000
C	1.44802300	3.51713000	-1.18305900
C	0.13120500	4.11529900	-0.63550700
C	-0.83761700	4.60267000	-1.71392000
C	-2.22060200	4.92559100	-1.13963400
C	-2.76096700	3.78881500	-0.26192100
C	-1.70595800	3.44034600	0.80405600
C	-5.65999000	-0.42890400	1.73439200
C	-5.72555100	1.09311600	1.82536800
C	-4.00311500	-5.04427200	-1.86759800
C	-1.05270900	-3.22626600	-2.70070300
C	0.84672700	-4.77736400	-0.05667000
C	3.88132700	-3.11856900	2.05438500
C	3.46974800	-4.47242600	2.62894200
C	4.93414300	-2.93112300	-1.92991900
C	6.33646800	1.79697400	-0.91731000
C	2.28370900	4.59954100	-1.85149200
C	-4.08910600	4.17391600	0.41576200
C	-5.26929800	4.27162200	-0.54649000
C	-1.48847800	4.47915700	1.90034100
O	-3.29862800	-2.99359000	0.28289300
O	-0.47194100	-1.29026100	-0.50357400
O	1.23059500	-2.03349500	1.79318600
O	4.09085800	-0.61250300	0.94090600
O	3.56366100	1.17887400	-0.48077400
O	2.19563900	3.01157700	-0.04961900
O	-0.47794500	3.09834600	0.15282900
O	-2.93977600	2.65912300	-1.10708700
O	0.76462700	1.52983500	1.87956000
H	-4.76473500	-0.51165200	-0.22262200
H	-4.63332400	-2.82917500	1.74782400
H	-5.79854800	-4.12052300	0.14266500
H	-6.63809700	-2.57537900	0.12946900
H	-5.42816600	-1.87444600	-1.91038000
H	-6.18925900	-3.43624800	-2.19454900
H	-3.81847300	-3.24383600	-3.02454300
H	-2.99196700	-1.75868100	-1.34948400
H	-1.57354100	-4.42578600	-0.98990400

H	-1.00627400	-2.84769300	0.75010200
H	1.33069900	-3.01411900	-1.20437700
H	3.36781500	-3.68939100	0.04640600
H	3.12258300	-1.23524100	-0.76378200
H	5.87411800	-2.33161600	-0.08236500
H	5.00992900	-0.25152800	-2.14774500
H	6.54562900	0.15789100	0.45909700
H	2.06772400	0.05757300	1.41838600
H	0.30634400	0.92636500	-0.66243000
H	2.10096100	2.18635200	-2.75459700
H	0.33504700	2.31549000	-2.67014000
H	0.40803000	4.95417100	0.01998400
H	-0.42112700	5.49006100	-2.20158000
H	-0.95572400	3.82982200	-2.47750700
H	-2.91800400	5.10887300	-1.96388000
H	-2.17983900	5.84576300	-0.54504400
H	-2.01069900	2.49933400	1.27546200
H	-5.46789900	-0.84931000	2.72898900
H	-6.63297400	-0.81376500	1.40742000
H	-5.87317400	1.54228600	0.83592900
H	-4.79888500	1.50041100	2.24611100
H	-6.55135200	1.41584400	2.46886900
H	-4.87152200	-5.48277500	-2.37273200
H	-3.10475700	-5.46771000	-2.32975500
H	-4.02309700	-5.36621700	-0.82101400
H	-0.04267700	-3.63272700	-2.81698700
H	-1.69929500	-3.76927300	-3.39634900
H	-1.02838500	-2.17474100	-3.00686600
H	1.85631100	-5.16359200	0.10788400
H	0.44649100	-5.27818000	-0.94201100
H	0.23319900	-5.05680800	0.80725600
H	3.62572900	-2.31718400	2.75293700
H	4.96916200	-3.09211600	1.92552800
H	3.74510600	-5.28987100	1.95184400
H	2.38737800	-4.52670700	2.79573300
H	4.11929000	-2.60744800	-2.59025200
H	5.84282800	-3.01251400	-2.53720700
H	4.69501400	-3.93464400	-1.56350900
H	7.27722000	1.61294100	-1.44845600
H	6.52872200	2.56669200	-0.16272900
H	5.61217500	2.19539000	-1.63499100
H	1.80747700	4.94635800	-2.77392400
H	2.41964800	5.45796100	-1.18519800
H	3.26791800	4.19380800	-2.10307900
H	-4.31135000	3.41975200	1.18104400
H	-3.96391600	5.12704900	0.94317700

H	-6.19847000	4.45727400	0.00353700
H	-5.38288500	3.34205700	-1.11183700
H	-5.14163600	5.08935900	-1.26501600
H	-0.62753400	4.18713300	2.51152600
H	-2.36243300	4.52366500	2.55625700
H	-1.30516300	5.48466800	1.50994300
H	-1.35145800	-0.86297700	-0.43446900
H	-2.89271000	1.84428100	-0.56730100
H	0.44670500	2.28709100	1.33826500
H	3.96215300	-4.65325700	3.59069000
C	3.15090600	1.66424500	2.33537200
H	2.92724100	2.70652300	2.58017600
H	3.10745000	1.09187900	3.26668400
H	6.66220500	-0.77678900	-1.82245100
O	-2.51231700	0.24787700	0.35753000
C	4.54534200	1.55756300	1.68468400
H	4.88329200	2.54436200	1.35915700
H	5.28613700	1.16543700	2.38677200
F	1.69756700	-0.02426500	-1.74335800
O	-2.76669600	-0.71817300	2.34860400
Na	-0.52560100	-0.35160800	1.73921500

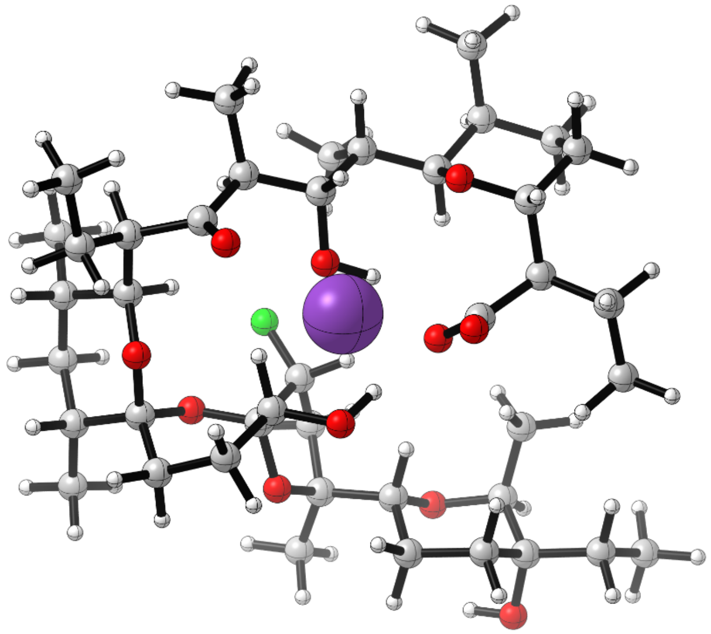

Figure S27. Structure of [K]$^{+}$-**4** optimized at the (SMD = MeCN) ωB97X-D/6-31G(d) level of theory.

Zero-point correction=	1.111330 (Hartree/Particle)
Thermal correction to Energy=	1.168478

Thermal correction to Enthalpy=	1.169422
Thermal correction to Gibbs Free Energy=	1.023398
Sum of electronic and zero-point Energies=	-3168.253034
Sum of electronic and thermal Energies=	-3168.195886
Sum of electronic and thermal Enthalpies=	-3168.194942
Sum of electronic and thermal Free Energies=	-3168.340966

Number of Imaginary Frequencies = 0

Charge = 0, Multiplicity = 1

C	2.16740200	1.81020700	1.24902900
C	3.06309700	2.88587900	0.63047700
C	2.26512400	4.21150800	0.58524500
C	2.91652600	5.28883500	-0.28402600
C	2.73982800	4.97069200	-1.76769700
C	1.25666600	4.77460300	-2.11581300
C	0.67095600	3.69454000	-1.17871400
C	-0.84710800	3.48092400	-1.35951900
C	-1.47293900	2.69772800	-0.18850000
C	-2.96685200	2.41699600	-0.37478400
C	-3.53454900	1.55288700	0.74329700
C	-4.71877400	0.66696900	0.37099400
C	-4.20889200	-0.63894200	-0.28576500
C	-5.28152600	-1.37368200	-1.10642600
C	-4.75602700	-2.76020000	-1.49973000
C	-4.21377900	-3.55057200	-0.30386000
C	-3.15099800	-2.69813200	0.40356900
C	-0.90245600	-1.41046100	1.23945900
C	-0.82431500	-2.13780600	-0.11137500
C	-0.22033700	-1.34466500	-1.30159000
C	0.49083400	-2.42336600	-2.09787400
C	1.01872400	-3.33629400	-0.98992000
C	2.31948300	-2.73966300	-0.41145800
C	2.78505500	-3.30650700	0.92143400
C	4.09983600	-2.62068400	1.30605500
C	5.13775900	-2.71501200	0.17312000
C	4.53137900	-2.17903400	-1.14245900
C	4.35363600	3.04200100	1.44687600
C	5.20102800	1.77071500	1.47246300
C	0.48527200	6.09804500	-2.06578100
C	-1.12602800	2.81558200	-2.71078200
C	-3.80444900	3.70591500	-0.42772300
C	-5.65162800	0.43578400	1.56194500
C	-6.40514400	1.70280400	1.96318200
C	-5.68926300	-0.58459800	-2.35035800
C	-3.70237900	-4.92539500	-0.72725400

C	1.16827000	-4.78893600	-1.40602200
C	6.43459000	-2.00885200	0.57214200
C	7.53653200	-2.03748700	-0.48506600
C	4.32634600	-0.67047400	-1.22780900
O	0.90010000	4.02304200	0.19183000
O	-0.86752000	1.43263100	0.00470900
O	-3.10603200	1.60806300	1.88629100
O	-3.72285600	-1.45413400	0.77374500
O	-2.10869200	-2.52263500	-0.54140600
O	0.00857700	-3.26623600	0.03307000
O	3.32664700	-2.90735700	-1.40748800
O	5.46645800	-4.09074800	-0.04469700
O	0.35636100	-1.03289300	1.75469700
H	3.31794600	2.56404900	-0.38486800
H	2.18368700	4.56942100	1.61699500
H	2.44567800	6.25141200	-0.05222600
H	3.97716300	5.38967200	-0.03393000
H	3.29512100	4.05858300	-2.02152000
H	3.16176600	5.77629000	-2.37988700
H	1.20776800	4.38671700	-3.13959700
H	1.18526700	2.74663000	-1.40590500
H	-1.32049300	4.46817900	-1.34065100
H	-1.35413200	3.31300400	0.71546400
H	-3.11592400	1.86496700	-1.30886700
H	-5.26353400	1.20427100	-0.41433800
H	-3.37102600	-0.40699400	-0.95773900
H	-6.16379000	-1.51844300	-0.46894600
H	-3.95733800	-2.64710300	-2.24495600
H	-5.02524600	-3.67888400	0.42481700
H	-1.52719200	-0.53271200	1.05535300
H	0.45648000	-0.56178300	-0.95835500
H	-0.24616400	-2.95139800	-2.71214800
H	1.28919700	-2.04053700	-2.73608900
H	2.14620500	-1.66708800	-0.25656300
H	2.01535600	-3.11630500	1.67278100
H	2.93145100	-4.39113000	0.84944200
H	4.52685100	-3.07722300	2.20631600
H	3.90454400	-1.56487200	1.53648100
H	5.19791600	-2.48299200	-1.95615200
H	4.08164100	3.32529200	2.47041600
H	4.95672600	3.85933600	1.03588000
H	5.51848600	1.49024700	0.46190400
H	4.64359700	0.92670600	1.89643300
H	6.10193600	1.91253600	2.07984400
H	1.05892800	6.89277900	-2.55650200
H	-0.47782700	6.02495400	-2.58148900

H	0.29004300	6.41193300	-1.03459900
H	-0.55633700	3.28497800	-3.51806700
H	-0.86082300	1.75353800	-2.67628300
H	-2.18397700	2.89174300	-2.98128500
H	-3.60491500	4.33042900	0.45039300
H	-4.87675700	3.48613300	-0.45276800
H	-3.56540200	4.28301800	-1.32562200
H	-5.06286900	0.06366500	2.40475900
H	-6.37403700	-0.34714400	1.30818200
H	-7.02839300	2.06805000	1.13792200
H	-5.71564600	2.50710200	2.24435100
H	-4.80850200	-0.32357400	-2.95104800
H	-6.35571600	-1.18584200	-2.97922900
H	-6.21907500	0.34159200	-2.10476900
H	-4.48346500	-5.46567500	-1.27412500
H	-3.41617000	-5.53922900	0.13316200
H	-2.83056000	-4.82796200	-1.38242400
H	1.93482700	-4.88630800	-2.17955700
H	1.45409300	-5.41404400	-0.55408300
H	0.21744700	-5.16000300	-1.80105300
H	6.19987100	-0.97304900	0.84487400
H	6.79683300	-2.49709000	1.48601000
H	8.46294500	-1.61283400	-0.08332900
H	7.26756500	-1.45625300	-1.37423400
H	7.74177500	-3.06463000	-0.80199100
H	3.78827000	-0.43588000	-2.15307200
H	5.29585300	-0.16414200	-1.26595300
H	3.75557000	-0.25470400	-0.39351000
H	0.10907500	1.49325800	-0.01173500
H	4.73288200	-4.45897200	-0.56017300
H	0.81714400	-0.36084900	1.19073900
H	-7.06145500	1.51305200	2.81966700
C	-1.56401700	-2.30422200	2.29678100
H	-0.77241000	-2.86621200	2.80000100
H	-2.04072400	-1.66422600	3.04676900
H	-5.56278800	-3.32967700	-1.97750000
O	1.90143200	1.88705700	2.46558700
C	-2.58969300	-3.27539600	1.69282300
H	-2.10726800	-4.22573400	1.45229500
H	-3.40728500	-3.47752500	2.39051900
F	-1.20124200	-0.71818800	-2.05692300
O	1.71608500	0.91337700	0.45592200
K	-0.62774300	1.35602300	2.82621400

References

[1] F. Ghorbani, S. A. Harry, J. N. Capilato, C. R. Pitts, J. Joram, G. N. Peters, J. D. Tovar, I. Smajlagic, M. A.Siegler, T. Dudding and T. Lectka, *J. Am. Chem. Soc.*, 2020, **142**, 14710-14724.

[2] F. G. Riddell and S. J. Tompsett. *Tetrahedron*, 1991, **47**, 10109-10118.

[3] A. Versini, L. Colombeau, A. Hienzsch, C. Gaillet, P. Retailleau, S. Debieu, S. Müller, T. Cañeque and R. Rodriguez, *Chem. Eur. J.*, 2020, **26**, 7416 – 7424.

[4] H. Asukabe, H. Yoneyama, Y. Mori, K.-I. Harada and M. Suzuki, *J. Chromatogr.,* 1987, **397**, 261-271.

[5] H. Luhavaya, S. R. Williams, H. Hong, L. Gonzaga de Oliveira, and P. F. Leadlay, *ChemBioChem*, 2014, **15**, 2081-2085.

[6] C. Thibaudeau, J. Plavec, and J. Chattopadhyaya, *J. Org. Chem.*, 1998, **63**, 4967-4985.

[7] Schrödinger Release 2022-3: MacroModel, Schrödinger, LLC, New York, NY, 2021.

[8] Gaussian 16, Revision C.01, Frisch, M. J.; Trucks, G. W.; Schlegel, H. B.; Scuseria, G. E.; Robb, M. A.; Cheeseman, J. R.; Scalmani, G.; Barone, V.; Petersson, G. A.; Nakatsuji, H.; Li, X.; Caricato, M.; Marenich, A. V.; Bloino, J.; Janesko, B. G.; Gomperts, R.; Mennucci, B.; Hratchian, H. P.; Ortiz, J. V.; Izmaylov, A. F.; Sonnenberg, J. L.; Williams-Young, D.; Ding, F.; Lipparini, F.; Egidi, F.; Goings, J.; Peng, B.; Petrone, A.; Henderson, T.; Ranasinghe, D.; Zakrzewski, V. G.; Gao, J.; Rega, N.; Zheng, G.; Liang, W.; Hada, M.; Ehara, M.; Toyota, K.; Fukuda, R.; Hasegawa, J.; Ishida, M.; Nakajima, T.; Honda, Y.; Kitao, O.; Nakai, H.; Vreven, T.; Throssell, K.; Montgomery, J. A., Jr.; Peralta, J. E.; Ogliaro, F.; Bearpark, M. J.; Heyd, J. J.; Brothers, E. N.; Kudin, K. N.; Staroverov, V. N.; Keith, T. A.; Kobayashi, R.; Normand, J.; Raghavachari, K.; Rendell, A. P.; Burant, J. C.; Iyengar, S. S.; Tomasi, J.; Cossi, M.; Millam, J. M.; Klene, M.; Adamo, C.; Cammi, R.; Ochterski, J. W.; Martin, R. L.; Morokuma, K.; Farkas, O.; Foresman, J. B.; Fox, D. J. Gaussian, Inc., Wallingford CT, 2016.

[9] J.-D. Chai, M. Head-Gordon, *Phys. Chem. Chem. Phys.* **2008**, *10*, 6615.

[10] A. V. Marenich, C. J. Cramer, D. G. Truhlar, *The Journal of Physical Chemistry B.* **2009**, *113*, 6378.

[11] Gagliardi, L. G.; Castells, C. B.; Ràfols, C.; Rosés, M.; Bosch, E. *J. Chem. Eng. Data*. **2007,** 52, 1103.

[12] Schrödinger Release 2022-3: Jaguar, Schrödinger, LLC, New York, NY, 2021.

[13] Legault, C. Y. CYLview, version 1.0b; Université de Sherbrooke: Quebec, Canada, **2009**; http://www.cylview.org.

[14] Dennington, R.; Keith, T. A.; Millam, J. M. GaussView, Version 6.1; Semichem Inc., Shawnee Mission, KS, 2016.
